# Functional Characterization of an In-Frame Deletion in the Basic Domain of the Retinal Transcription Factor ATOH7

**DOI:** 10.3390/ijms23031053

**Published:** 2022-01-19

**Authors:** David Atac, Lucas Mohn, Silke Feil, Kevin Maggi, Dominik Haenni, Britta Seebauer, Samuel Koller, Wolfgang Berger

**Affiliations:** 1 Institute of Medical Molecular Genetics, University of Zurich, 8952 Schlieren, Switzerland; grubichatac@medmolgen.uzh.ch (D.A.); lucas.mohn@uzh.ch (L.M.); feil@medmolgen.uzh.ch (S.F.); kmaggi@medmolgen.uzh.ch (K.M.); b.seebauer@gmx.ch (B.S.); koller@medmolgen.uzh.ch (S.K.); 2 Neuroscience Center Zurich, University and ETH Zurich, 8057 Zurich, Switzerland; 3 Center for Microscopy and Image Analysis, University of Zurich, 8057 Zurich, Switzerland; dominik.haenni@bluewin.ch; 4 Zurich Center for Integrative Human Physiology, University of Zurich, 8057 Zurich, Switzerland

**Keywords:** atonal bHLH transcription factor 7 (*ATOH7*), basic helix–loop–helix (bHLH), cycloheximide (CHX) chase assay, protein dimerization, synergistic nuclear import, dimerization-mediated proteasomal degradation, DNA-binding, fluorescence lifetime imaging-Förster resonance energy transfer (FLIM–FRET), nonsyndromic congenital retinal nonattachment (NCRNA), retina

## Abstract

Basic helix–loop–helix (bHLH) transcription factors are evolutionarily conserved and structurally similar proteins important in development. The temporospatial expression of atonal bHLH transcription factor 7 (*ATOH7*) directs the differentiation of retinal ganglion cells and mutations in the human gene lead to vitreoretinal and/or optic nerve abnormalities. Characterization of pathogenic *ATOH7* mutations is needed to understand the functions of the conserved bHLH motif. The published *ATOH7* in-frame deletion p.(Arg41_Arg48del) removes eight highly conserved amino acids in the basic domain. We functionally characterized the mutant protein by expressing V5-tagged *ATOH7* constructs in human embryonic kidney 293T (HEK293T) cells for subsequent protein analyses, including Western blot, cycloheximide chase assays, Förster resonance energy transfer fluorescence lifetime imaging, enzyme-linked immunosorbent assays and dual-luciferase assays. Our results indicate that the in-frame deletion in the basic domain causes mislocalization of the protein, which can be rescued by a putative dimerization partner transcription factor 3 isoform E47 (E47), suggesting synergistic nuclear import. Furthermore, we observed (i) increased proteasomal degradation of the mutant protein, (ii) reduced protein heterodimerization, (iii) decreased DNA-binding and transcriptional activation of a reporter gene, as well as (iv) inhibited E47 activity. Altogether our observations suggest that the DNA-binding basic domain of ATOH7 has additional roles in regulating the nuclear import, dimerization, and protein stability.

## 1. Introduction

Basic helix–loop–helix (bHLH) proteins are structurally similar dimerizing transcription factors with a crucial role in development. Monomer dimerization is required for protein function and occurs through interactions mediated by the HLH domain, which is formed by two conserved alpha helixes interconnected by a non-conserved loop [1,2,3]. The formed dimer mediates transcriptional activation by interacting with DNA through the basic domain. The target DNA motif of bHLH proteins is a pseudo-palindromic DNA-binding enhancer box (E-box) consisting of the consensus sequence CANNTG.

Classification of bHLH transcription factors can be made based on their expression pattern [3]. Class I bHLH transcription factors, also known as E proteins, are ubiquitously expressed. All members are expressed by the E protein gene family, consisting of transcription factor 3 (*TCF3*; OMIM: * 147141)-encoded isoforms E12 (RefSeq: NM_003200.5) and E47 (RefSeq: NM_001351779.2), transcription factor 4 (*TCF4*; OMIM: * 602272) and transcription factor 12 (*TCF12*; OMIM: * 600480). Amongst these, the crystal structure of E47 was determined within a DNA-bound dimer involving the bHLH transcription factor neuronal differentiation 1 (NEUROD1; OMIM: * 601724) [4]. On the other hand, class II bHLH transcription factors exhibit a time-limited and tissue-specific expression pattern and usually require heterodimerization with E proteins in order to exert their function [3]. Class II bHLH transcription factors are involved in the development of various tissues, including muscle, blood, and fly sex determination [5,6,7].

Class II bHLH transcription factors are also crucial in retinogenesis, as members of this class determine the cell fate of retinal progenitor subpopulations [8,9,10,11,12,13]. An example is the evolutionary conserved atonal bHLH transcription factor 7 (*ATOH7*; OMIM: * 609875). The single-exon *ATOH7* gene encodes a protein of 152 amino acids where positions 41–52 form the basic domain and positions 53–93 form the HLH domain [14,15,16]. Expression of *Atoh7* in studied vertebrates, where it is also known as mouse atonal homolog 5 (*Math5*; RefSeq: NM_016864), Xenopus laevis atonal homolog 5 (*Xath5*; RefSeq: NM_001085821), zebrafish atonal homolog 5 (*Zath5*; RefSeq: NM_131632) and chicken atonal homolog 5 (*Cath5*; RefSeq: NM_204668), were shown to closely coincide with the differentiation of retinal ganglion cells (RGCs) [9,17,18,19,20,21]. The relevance of this transcription factor in eye development is well exemplified in mouse and zebrafish knockout models, which show a complete lack of RGCs and therefore absent optic nerves, as well as aberrations of the retinal vasculature [22,23].

To this date, several mutations in *ATOH7* have been associated with human eye disorders (Table 1) leading to a spectrum of vitreoretinal traits, including persistent hyperplastic primary vitreous (PHPVAR; OMIM: #221900), nonsyndromic congenital retinal nonattachment (NCRNA; OMIM: #221900), familial exudative vitreoretinopathy (EVR; OMIM: #133780), as well as bilateral optic nerve hypoplasia (ONH; OMIM: #165550) [24,25,26,27,28,29]. Functional characterization of the NCRNA-associated basic domain mutation p.(Asn46His), hereinafter ATOH7:N46H, revealed decreased DNA-binding, loss of transcriptional activation and the inability to rescue RGC differentiation in mouse *Atoh7^−/−^* retinal explants [26]. In the same study, two ONH-associated ATOH7 variants were investigated: the basic domain mutation p.(Ala47Thr) retained partial function and was deemed to be a hypomorphic allele, while the HLH domain variant p.(Arg65Gly) was shown to be fully functional and thus not likely causative of the phenotype [26,30]. Furthermore, protein analyses by our lab established that the ONH-associated HLH missense mutations p.(Ala59Thr) and p.(Ala59Val), hereinafter ATOH7:A59T and A59V, lead to dimerization-mediated proteasomal degradation in the presence of the putative dimerization partner E47, weakened protein dimerization, reduced DNA-binding, and resulted in a complete loss of transcriptional activation [29].

Further functional analyses of pathogenic *ATOH7* mutations are needed to fully understand the fundamental functions of the conserved bHLH motif in ATOH7 and to pinpoint the mechanisms behind the pleiotropy of the associated eye disorders. Consequently, in this article we report the functional characterization of a previously published NCRNA-associated in-frame deletion in *ATOH7*, NM_145178.4:c.121_144del; p.(Arg41_Arg48del), hereinafter ATOH7:R41_R48del [27]. This deletion removes eight amino acids within the basic domain, of which seven are evolutionarily conserved in *Drosophila melanogaster*. In addition to the expected decrease in DNA-binding and the loss of transcriptional reporter gene activation, the mutant ATOH7:R41_R48del protein presented subcellular mislocalization, increased proteasomal degradation, reduced heterodimerization, as well as functional inhibition of the putative dimerization partner E47. We also observed rescued subcellular localization of the mutant ATOH7:R41_R48del protein when co-expressed with E47, suggesting synergistic nuclear import: a sparsely reported but potentially important characteristic of bHLH transcription factors. Altogether these observations suggest that the DNA-binding basic domain of ATOH7 has additional functions in regulating the nuclear import, dimerization, and protein stability.

## 2. Results

### 2.1. The NCRNA-Associated In-Frame Deletion R41_R48del Removes Evolutionary Conserved Amino Acids in the Basic Domain of ATOH7

**Figure 1:**
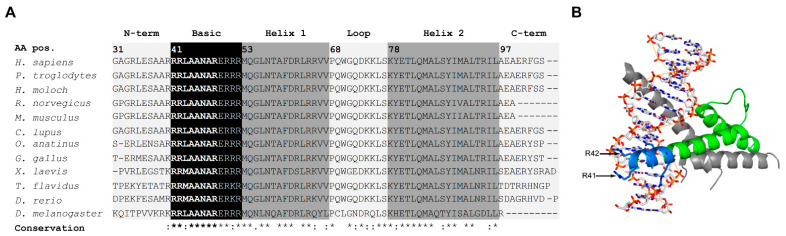
The in-frame deletion ATOH7:R41_R48del predicts the removal of evolutionarily conserved amino acids within the basic domain of ATOH7. (**A**) MSA of bHLH protein sequences of twelve ATOH7 homologs. Seven out of the eight deleted amino acids (in bold) are highly conserved between the investigated species. (**B**) A three-dimensional model of the bHLH structural motif of ATOH7 (blue, deleted basic domain positions Arg41–Arg48; green, remaining bHLH motif) dimerized with the transcription factor E47 (grey) and bound to dsDNA. The deleted amino acids within the ATOH7 basic domain are in close connection to the major groove of the DNA helix. Asterisk (*), fully conserved amino acid residue; colon (:), amino acid residues with strongly similar properties; period (.), amino acid residues with weakly similar properties. AA pos, amino acid position; ATOH7, atonal bHLH transcription factor 7; bHLH, basic helix–loop–helix; C-term, C-terminus; dsDNA, double-stranded DNA; E47, transcription factor 3 isoform E47; MSA, multiple sequence alignment; NCRNA, nonsyndromic congenital retinal nonattachment; N-term, N-terminus.

As previously described by Kondo et al., the homozygous in-frame deletion of 24 bp detected in a three-month-old boy diagnosed with NCRNA predicts the removal of eight amino acids (Arg41 to Arg48) within the highly conserved ATOH7 basic domain [27]. The evolutionary conservation of the basic domain was evaluated by multiple sequence alignment (MSA) of twelve ATOH7 homologs (Figure 1A). To illustrate the binding of an ATOH7 heterodimer to DNA, the reference protein structure was modeled based on the crystallized structure of a DNA-bound dimer between the class I bHLH transcription factor E47 (RefSeq: NM_001351779.2) and the ATOH7-related class II bHLH transcription factor NEUROD1 (RefSeq: NM_010894) [4]. The modeling confirmed close interaction of the deleted ATOH7 amino acids with the DNA major groove (Figure 1B).

### 2.2. The Mutant ATOH7:R41_R48del Protein Is Misslocalized Due to the Loss of a Predicted NLS within the Basic Domain

**Figure 2:**
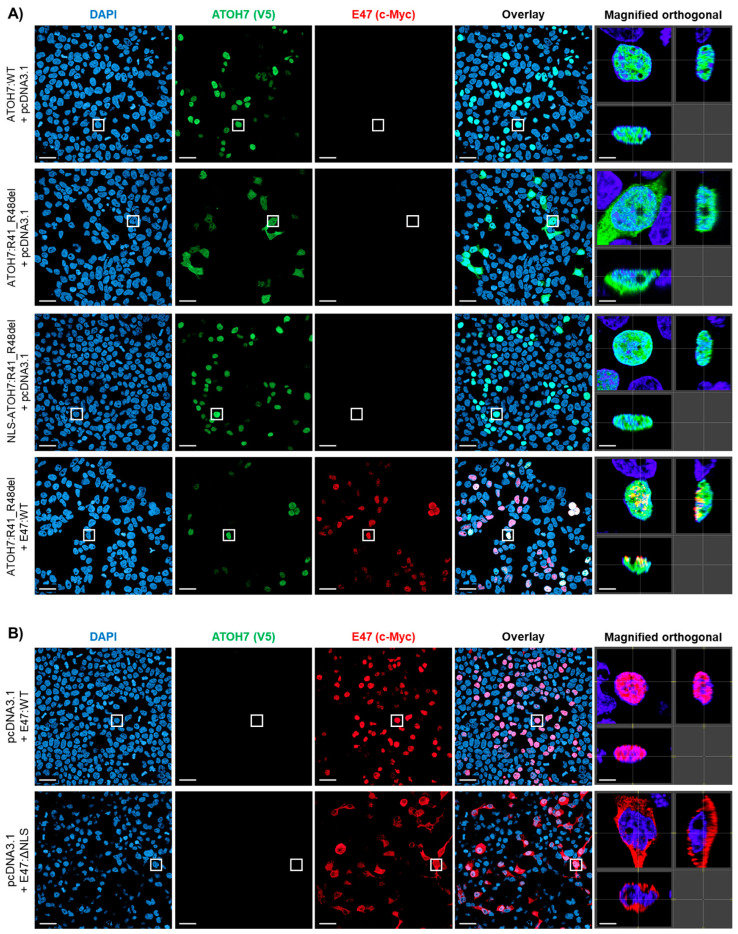

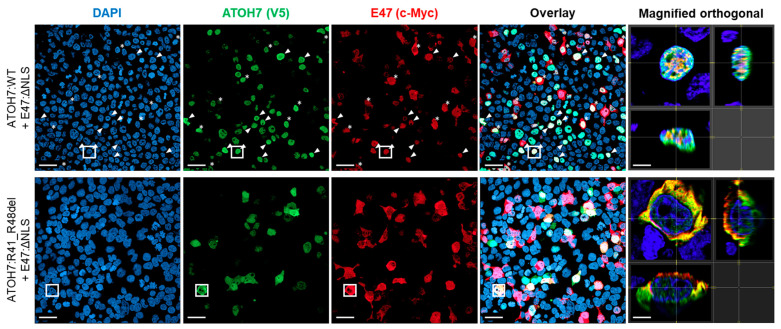
The mutant ATOH7:R41_R48del protein is mislocalized due to the loss of a predicted NLS within the basic domain. Confocal immunofluorescence microscopy images showing the subcellular localization of V5-tagged ATOH7 (green) and c-Myc-tagged E47 (red) proteins expressed in HEK293T cells after 4h of proteasomal inhibition by MG-132 (images without proteasomal inhibition are shown in Figure S2). Nuclei are stained by DAPI (blue). Boxes mark the position of example cells shown as XY, XZ and YZ projections (magnified orthogonal; separate channels are shown in Figure S3). Scale bars represent 30 µm (overview image) and 5 µm (magnified orthogonal). (**A**) Complete rescue of the mislocalized ATOH7:R41_R48del protein by the presence of an artificial N-terminal NLS sequence derived from the SV40 large T-antigen or by co-transfection with E47:WT, respectively. (**B**) Partial rescue of the mislocalized E47:ΔNLS variant by ATOH7:WT. Arrowheads point at co-expressing cells with complete rescue of the subcellular localization of E47:ΔNLS protein, while asterisks (*) mark co-expressing cells with no or partial rescue. ATOH7, atonal bHLH transcription factor 7; DAPI; 4′,6-diamidino-2-phenylindole; E47, transcription factor 3 isoform E47; HEK293T, human embryonic kidney 293T; NLS, nuclear localization signal; SV-40, simian vacuolating virus 40; WT, wild-type.

Plasmid constructs carrying V5-tagged *ATOH7* coding sequences (RefSeq: NM_145178.4) were expressed in human embryonic kidney 293T (HEK293T) cells and visualized by confocal immunofluorescence microscopy. The mutant ATOH7:R41_R48del protein revealed a pancellular expression pattern with strong extranuclear retention, in sharp contrast to the expected nuclear localization of the wild-type (WT) protein (Figure S2). In order to increase the protein levels of ATOH7, confocal microscopy was also performed after treatment of HEK293T cells with the proteasomal inhibitor MG-132, which highlighted the pancellular localization of ATOH7:R41_R48del, while the localization of the WT protein remained nuclear (Figure 2A). These findings were consistent with the presence of a nuclear localization signal (NLS; Figure S1A) predicted within the ATOH7 basic ATOaasbasic domain by the NLS prediction algorithm NLStradamus [32] (Alan Moses’ Lab, University of Toronto, Toronto, Canada; http://www.moseslab.csb.utoronto.ca/NLStradamus; accessed on 18 August 2020). Conversely, the addition of an N-terminal NLS derived from the simian vacuolating virus 40 (SV-40) large T-antigen (Pro-Lys-Lys-Lys-Arg-Lys-Val) was able to rescue the nuclear localization of the NLS-ATOH7:R41_R48del protein (Figure 2A and Figure S2).

Co-expression of c-Myc-tagged E47 protein (RefSeq: NM_001136139.4) was also able to restore the subcellular localization of ATOH7:R41_R48del protein, suggesting possible heterodimerization of the proteins in the cytoplasm and subsequent synergistic import into the nucleus utilizing the NLS of the E47 protein (Figure 2A and Figure S2). A mutant E47 protein p.(Arg158_Arg174del), hereinafter E47:ΔNLS, was created based on NLS sequence prediction by NLStradamus (Figure S1B) and showed mislocalization to the cytoplasm instead of being present exclusively in the nucleus (Figure 2B and Figure S2). The subcellular localization of E47:ΔNLS was fully restored in some cells by the co-expression of ATOH7:WT (Figure 2B and Figure S2). This finding was not observed when E47:ΔNLS was co-expressed with the ATOH7:R41_R48del mutant (Figure 2B).

### 2.3. Decreased Amount of the Mutant ATOH7:R41_R48del Protein Is the Result of an Increased Proteasomal Degradation

**Figure 3:**
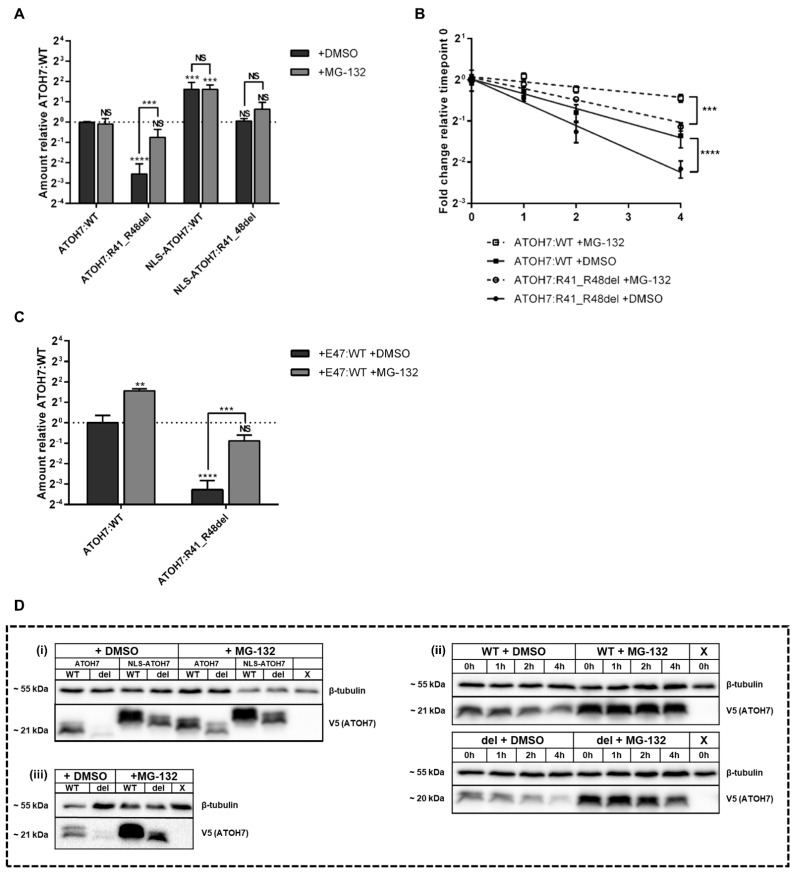
Decreased amount of the mutant ATOH7:R41_R48del protein is a result of increased proteasomal degradation. Plasmid constructs were expressed in HEK293T cells and cell lysates were subsequently analyzed by SDS-PAGE. (**A**) Semi-quantitative WB of V5-tagged ATOH7 normalized to β-tubulin and presented as fold-change relative to ATOH7:WT. The cells were treated with either DMSO or the proteasome inhibitor MG-132 for four hours before cell lysis. (**B**) CHX chase assay showing ATOH7 protein turnover rate quantified through semi-quantitative WB of V5-tagged ATOH7 normalized to β-tubulin and presented as fold-change relative to ATOH7:WT protein amount at timepoint zero. The cells were treated with either DMSO or the proteasome inhibitor MG-132 for four hours before adding CHX. (**C**) Semi-quantitative WB of V5-tagged ATOH7 normalized to β-tubulin and presented as fold-change relative to ATOH7:WT. The putative dimerization partner E47:WT was co-expressed. The cells were treated with either DMSO or the proteasome inhibitor MG-132 for four hours before cell lysis. (**D**) Cropped example blots for A–C represented by (i–iii), respectively. Full blots are shown in Figure S4. Each bar or point represents the mean ± SD of three separate experiments. **, *** and **** represent *p*-values of <10^−2^, <10^−3^ and <10^−4^, respectively. ATOH7, atonal bHLH transcription factor 7; CHX, cycloheximide; del, ATOH7:R41-R48del; DMSO, dimethyl sulfoxide; E47, transcription factor 3 isoform E47; HEK293T, human embryonic kidney 293T; NLS, nuclear localization signal; NS, not significant; SD, standard deviation; SDS-PAGE, sodium dodecyl sulfate polyacrylamide gel electrophoresis; WB, Western blot; WT, wild-type; X, empty backbone (pcDNA3.1).

Western blot (WB) of HEK293T cell lysates (Figure S4) revealed a significant reduction in protein amount of the mutant ATOH7:R41_R48del protein compared to WT, which was restored by inhibiting proteasomal degradation by MG-132 treatment (Figure 3A). The protein amount of the ATOH7:R41_R48del mutant was also restored by linking the protein to the SV40-derived NLS (NLS-ATOH7:R41_R48del), whereas the protein amount of ATOH7:WT linked to this artificial NLS (NLS-ATOH7:WT) was significantly increased compared to ATOH7:WT (Figure 3A,D). Protein amounts of the artificial NLS-linked constructs were not significantly affected by proteasomal inhibition (Figure 3A).

In order to verify whether the reduced protein amount of ATOH7:R41_R48del was caused by altered protein decay, cycloheximide (CHX) chase assay was performed (Figure 3B). Protein amounts of ATOH7 were quantified at timepoints 0–4 h post-CHX addition, revealing an increased proteasome-mediated turnover rate of the ATOH7:R41_R48del protein compared to ATOH7:WT.

To test whether the increased proteasomal degradation is primarily a result of extranuclear retention, nuclear transport of the ATOH7:R41_R48del protein was also stimulated by co-expressing E47:WT. The increased proteasome-dependent protein degradation of ATOH7:R41_R48del persisted, indicating that the subcellular localization alone is not sufficient to explain protein instability (Figure 3C). Interestingly, proteasomal inhibition also increased ATOH7:WT protein levels when co-expressing E47:WT (Figure 3C). In contrast, MG-132 had no effect on ATOH7:WT stability in mono-expression experiments (Figure 3A).

### 2.4. Reduced Nuclear Dimerization of Mutant ATOH7:R41_R48del Protein and Extranuclear Protein Interaction with E47 in Living Cells

#### 2.4.1. FLIM–FRET Confirms Dimerization of ATOH7 and E47 in Living Cells

The interaction between the ATOH7 and E47 proteins was quantified through Förster resonance energy transfer (FRET) in living HEK293T cells imaged by fluorescence lifetime imaging (FLIM). Standard, light microscopy-based FRET measurements result in apparent FRET efficiencies, where the measured transfer efficiency depends on the distance of the interacting donor–acceptor molecules, as well as on the fraction of acceptor-bound donors within the sample. In contrast, FLIM–FRET experiments rely on the fluorescence lifetime τ of the donor molecule, which allows disentanglement of those two components (in the case of a donor with a mono-exponential lifetime decay) [33,34,35]. The FLIM–FRET efficiency represents the actual FRET, i.e., the degree of distance-dependent quenching that the acceptor exerts on the donor fluorophore. Conversely, the fraction of the acceptor-interacting donor molecules is determined by the donor–acceptor binding ratio.

**Figure 4:**
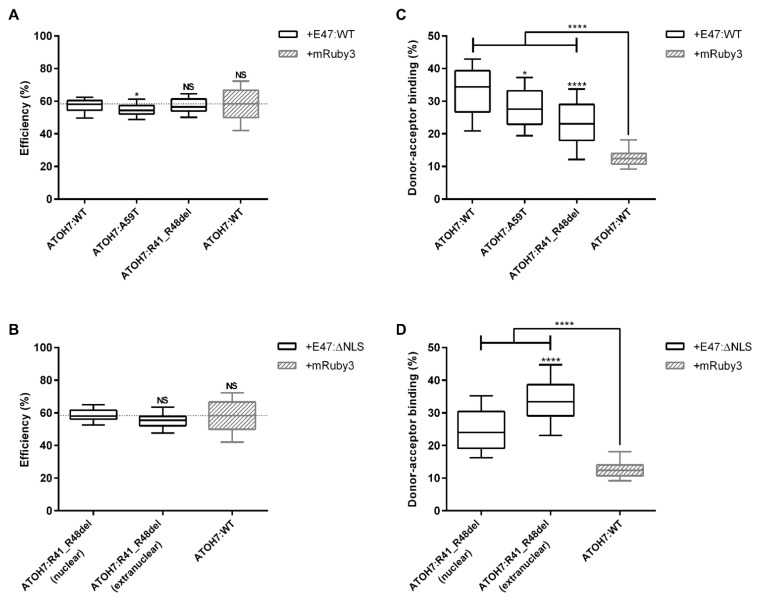
Reduced nuclear dimerization of mutant ATOH7:R41_R48del protein and possible cytosolic protein interaction with E47 in living cells. HEK293T cells were transfected with N-terminal mNeonGreen-linked ATOH7 donor and C-terminal mRuby3-linked E47 acceptor. Protein interaction was determined by TCSPC-based FLIM–FRET using an n-exponential reconvolution model to determine the fluorescence lifetime of the mono-exponential donor. (**A**,**B**) FLIM–FRET efficiencies were determined by calculating the ratio between the quenched and unquenched donor lifetimes. (**A**) Nuclear FLIM–FRET efficiencies of ATOH7:WT, the previously published HLH domain mutant ATOH7:A59T and the ATOH7:R41_R48del basic domain mutant. (**B**) Comparison of nuclear and extranuclear FLIM–FRET efficiencies of the ATOH7:R41_R48del mutant with a mislocalized NLS-deficient E47 protein E47:ΔNLS. (**C**,**D**) Donor–acceptor binding ratios. Amplitudes of fitted unbound and acceptor–bound donor species were determined and the donor–acceptor binding ratio was calculated (ratio of acceptor–bound donor relative to the sum of all donor species). (**C**) Nuclear donor–acceptor binding ratio of ATOH7:WT, A59T and R41_R48del. (**D**) Comparison of nuclear and extranuclear FLIM–FRET efficiencies ATOH7:R41_R48del mutant with E47:ΔNLS. Dotted line represents the level of the negative control, consisting of samples co-expressing ATOH7:WT donor constructs and free mRuby3 acceptor. Boxplots represent medians ± quartiles and 10–90 percentile whiskers of three independent experiments and at least 38 individual cells. * and **** represent *p*-values of <0.05 and <10^−4^, respectively. ATOH7, atonal bHLH transcription factor 7; E47, transcription factor 3 isoform E47; HEK293T, human embryonic kidney 293T; FLIM, fluorescence lifetime imaging; FRET, Förster resonance energy transfer; NLS, nuclear localization signal; NS, not significant; TCSPC, time-correlated single-photon counting; WT, wild-type.

Cells were transfected with mNeonGreen-linked ATOH7 donor and mRuby3-linked E47 acceptor expression constructs (Table S1). Time correlated single photon counting (TCSPC) histograms were fitted by an n-exponential reconvolution model. Due to a very good fit with evenly distributed residuals, the (ATOH7:WT) donor-only decay was determined to be mono-exponential (Figure S5A). The unquenched donor (UD) fluorescence lifetime of ATOH7:WT was 2.75 ± 0.14 ns (Figure S6A), whereas co-expression with the E47:WT acceptor resulted in shortened decays, indicating the presence of a FRET population with a quenched donor (QD) lifetime of 1.10 ± 0.20 ns (Figure S6C) and a calculated FLIM–FRET efficiency of 58.1 ± 5.9% (Figure 4A).

#### 2.4.2. Establishing a Background Threshold for Significant Protein Interaction

FLIM–FRET measurements of a negative control consisting of the ATOH7:WT donor co-expressed with a free mRuby3 acceptor showed a marginally shortened TCSPC decay compared to the donor-only control (Figure S6A,B) and was thus utilized as a background threshold in all statistical analysis (Figure 4 and Figure S6). It is possible that potential unspecific interactions between either mNeonGreen and mRuby3 fluorophores or between the ATOH7:WT protein and free mRuby3 occur in the living cell. Establishing the nature of these interactions is however outside the scope of this work. Nevertheless, the interaction of ATOH7:WT donor with E47:WT acceptor constructs resulted in a significantly higher percentage of acceptor-bound donors and apparent FRET efficiency compared to this negative control (Figure 4 and Figure S6D). Additionally, a relatively larger spread in donor–acceptor distance was seen in the negative control, highlighting the low specificity of its interaction (Figure S6E).

A small but significant shortening in the fitted UD lifetimes was found for constructs with a high donor–acceptor binding fraction compared to the donor-only lifetime (Figure S6B). A possible explanation could be that the ATOH7 protein is interacting with other cellular factors, thus altering the donor lifetime in the absence of an acceptor. On the other hand, the slight shortening of the UD lifetime in samples with high binding fractions may also represent a fitting artifact resulting from a relatively larger QD fraction. The shift in UD lifetime was however minor and did not lead to consistent differences in the corresponding FLIM–FRET efficiencies or binding fractions (Figure 4).

#### 2.4.3. Measurements of Protein Interactions between ATOH7 and E47

As described, FLIM–FRET allows the disentanglement of the apparent FRET efficiency (Figure S6D) into its two main components, the FLIM–FRET efficiency (Figure 4A,B) and the donor–acceptor binding ratio (Figure 4C,D). The FLIM–FRET efficiency is proportional to the interaction distance of two interacting molecules. The published ATOH7:A59T variant has previously shown reduced protein heterodimerization and was therefore included in FLIM–FRET experiments as a control variant [29]. A small but significant decrease in FLIM–FRET efficiency was seen for the ATOH7:A59T donor compared to ATOH7:WT when interacting with the E47:WT acceptor, whereas no statistical difference in FLIM–FRET efficiency was seen for the ATOH7:R41_R48del donor (Figure 4A). In addition, no statistically significant difference in FLIM–FRET efficiency was observed for the interaction of the ATOH7:R41_R48del protein with the misslocalized E47:ΔNLS acceptor, neither inside nor outside of the nucleus (Figure 4B).

In contrast, significant differences were seen in donor–acceptor binding ratios of interacting ATOH7 donors. The binding ratio of the ATOH7:R41_R48del donor with the E47:WT acceptor was significantly reduced compared to the ATOH7:WT donor (Figure 4C). A significant reduction of protein interaction was also seen for ATOH7:A59T, which was in line with previous results indicating reduced dimerization [29]. In order to confirm the hypothesis of ATOH7–E47 interaction in the cytoplasm as a prerequisite for synergistic nuclear import of an ATOH7–E47 dimer, a mislocalized E47:ΔNLS acceptor was utilized. Co-expression of this acceptor with the ATOH7:R41_R48del donor resulted in extranuclear areas with concentrated fluorophore intensities and an increased donor–acceptor binding ratio compared to the nuclear interaction of these constructs (Figure 4D), indicating cytosolic retention of the mutant ATOH7 protein and likely interaction with E47 prior to the proposed synergistic nuclear import of this heterodimer.

#### 2.4.4. The Reduced Dimerization of Mutant ATOH7:R41_R48del Protein with E47 Is Not a Result of Altered Protein Amounts

Although fluorescence lifetime measurements are independent of donor amounts, the QD population will inevitably increase with an increasing acceptor–donor ratio (A:D ratio) due to an altered equilibrium between the interacting molecules [35]. Acceptor and donor fluorescence were thus determined in order to discriminate whether the observed differences in acceptor–donor binding are a result of altered A:D ratios. In most cases, acceptor–donor binding was reduced despite significantly increased A:D ratios, indicating an interaction disturbance despite stoichiometrically more favorable conditions (Figure S6F–H). The extranuclear fraction of the mutant ATOH7:R41_R48del donor interacting with the E47:ΔNLS acceptor showed a higher A:D ratio compared to the nuclear fraction and could potentially explain the increased acceptor–donor binding outside the nucleus. Additionally, the interaction of the previously described negative control (ATOH7:WT donor and free mRuby3 acceptor) may have been influenced positively by a higher A:D ratio (Figure S6H).

### 2.5. The Mutant ATOH7:R41_R48del Protein Shows Reduced DNA-Binding, Reduced Transcriptional Activation and Inhibition of E47 Mediated Transcription

**Figure 5:**
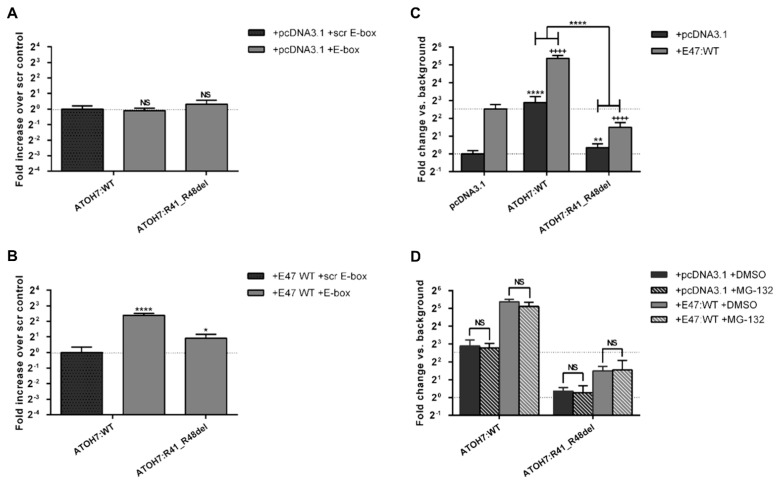
The mutant ATOH7:R41_R48del protein shows reduced DNA-binding, reduced transcriptional reporter activation and inhibition of the putative dimerization partner E47. (**A**,**B**) DNA-binding of ATOH7 was quantified through DNA-protein interaction ELISAs. V5-tagged ATOH7 from HEK293T cell lysate was mixed with lysate containing a dimerization partner consisting of either c-Myc-tagged ATOH7:WT (**A**) or c-Myc-tagged E47:WT (**B**) and was subsequently captured by biotin-streptavidin immobilized E-box DNA (7x CAGGTC). Results are normalized to background (sample without E-box DNA) and presented as fold-change relative to scr control (ATOH7:WT and scr E-box). Bars indicate mean of three independent experiments with three technical replicates ± SD. *, **, **** represent *p*-values of <0.05, <10^−2^ and <10^−4^, respectively. (**C**,**D**) Transcriptional activation of ATOH7 quantified through dual luciferase assay. HEK293T cells were transfected with plasmid firefly luciferase gene expressed under the control of an E-box-containing minimal promoter, renilla luciferase under the control of a CMV-promoter and pcDNA3.1 constructs containing ATOH7 or E47 under the control of a CMV-promoter. Firefly luminescence of each sample was normalized to renilla luciferase luminescence and is presented relative to controls transfected with an empty pcDNA3.1 vector. (**C**) Comparison of transcriptional activation induced by ATOH7:WT and R41_R48del proteins in absence and presence of E47:WT. (**D**) Comparison of transcriptional activation with or without a four-hour pretreatment with the proteasomal inhibitor MG-132. Bars indicate the mean of three independent experiments with four biological replicates ± SD. Light grey bars represent luminescence in absence of E47:WT; black bars represent luminescence in presence of E47:WT. Asterisk symbols (*) represent significance vs. control without E47 expression; cross symbols (+) represent significance vs. control with E47:WT expression. The 1–4 significance symbols represent *p*-values of <0.05, <10^−2^, <10^−3^ and <10^−4^, respectively. ATOH7, atonal bHLH transcription factor 7; CMV, cytomegalovirus; E-box, enhancer box; E47, transcription factor 3 isoform E47; ELISA, enzyme-linked immunosorbent assay; HEK293T, human embryonic kidney 293T; NLS, nuclear localization signal; NS, not significant; scr, scrambled; SD, standard deviation; WT, wild-type.

The DNA-binding ability of ATOH7 was tested by enzyme-linked immunosorbent assay (ELISA), as described in our previous publication [29]. In line with prior experience, no DNA-binding of ATOH7:WT protein could be observed in presence of the target E-box, unless the putative heterodimerization partner E47 was added (Figure 5A,B). In the presence of E47:WT, the mutant ATOH7:R41_R48del protein displayed a significantly decreased ability to bind DNA in comparison to WT, although binding was not completely abolished (Figure 5B). This observation is consistent with the position of the deleted amino acids, which are predicted to directly bind to the major groove of the DNA helix (Figure 1B).

Dual-luciferase assays of transfected HEK293T cells exhibited significantly reduced transcriptional reporter activation induced by the mutant ATOH7:R41_R48del protein compared to WT (Figure 5C). An interesting observation is that co-expression of E47:WT with ATOH7:R41_R48del reduced the transcriptional reporter activation below the baseline of E47 mono-expression. This is a striking and significant difference from our previously published ATOH7 loss-of-function mutations at amino acid position 59, located in the first helix domain of ATOH7, which did not reduce the transcriptional activation mediated by E47 [29].

Finally, in order to address whether the reduced transcriptional activation of mutant ATOH7 is due to abolished function of the protein rather than reduced protein amounts, the assay was replicated under proteasomal inhibition by MG-132 treatment (Figure 5D). No improvement of transcriptional activation was seen, confirming the impact of the mutation with respect to this particular protein function.

## 3. Discussion

### 3.1. Nuclear Localization of ATOH7 Is Directed by an NLS Contained within the Basic Domain and Can Also Be Stimulated by Synergistic Nuclear Import with E47

A bipartite NLS is commonly found within or around the basic domains of bHLH transcription factors [36,37,38,39,40]. A putative bipartite NLS was also predicted by NLStradamus to be located within the ATOH7 basic domain (Figure S1). The extranuclear retention of the mutant ATOH7:R41_R48del protein, which can be reversed by the addition of an N-terminal SV-40 NLS, provides evidence that an NLS is present within the basic domain (Figure 2A).

We also found compelling evidence for dimerization-mediated synergistic nuclear import of the mutant ATOH7:R41_R48del protein, as observed through immunocytochemistry (Figure 2A). Conversely, we noted an increased nuclear import of the mislocalized mutant E47:ΔNLS protein upon overexpression of ATOH7:WT (Figure 2B). These findings necessitate the interaction of the involved bHLH transcription factors outside the nucleus. Indeed, the acquired FLIM–FRET data support the hypothesis that ATOH7 and E47 may interact in the cytoplasm (Figure 4D and Figure S7), thereby enabling synergistic nuclear import. Although this is a novel finding concerning ATOH7, previously published research has shown that the atonal-related bHLH transcription factor NEUROD1 may be synergistically imported as a NEUROD1-E47 dimer in the absence of its NLS and vice versa [40]. The NEUROD1-E47 dimer import is energy-dependent and involves the Ran-mediated pathway [41,42]. Due to the structural and functional similarities with NEUROD1, it is possible that also ATOH7 heterodimers utilize a similar nuclear import mechanism.

### 3.2. Basic Amino Acid Residues in the ATOH7 Basic Domain May Be Involved in Regulating Protein Turnover through Proteasomal Degradation

A significantly increased turnover rate and decreased protein levels were observed for the mutant ATOH7:R41_R48del protein. It was proposed that the basically charged amino acids within the basic domain of the bHLH transcription factor nuclear receptor coactivator 3 (NCOA3; OMIM: * 601937) are involved in protein turnover, as substitution of these significantly alter the stability of the protein [38]. The observed significant increase in the turnover rate and resulting decrease in protein levels of the mutant ATOH7:R41_R48del protein (Figure 3A,B) could potentially also be attributed to the removal of the three basically charged arginines (Arg41, Arg42 and Arg48). Normalization of the mutant ATOH7:R41_R48del protein amounts, as well as a further increase in protein levels of ATOH7:WT by attachment of an N-terminal SV-40 NLS (Pro-Lys-Lys-Lys-Arg-Lys-Val) may indeed be a consequence of the addition of a lysine-rich basically charged peptide (Figure 3A,D).

It could be argued that subcellular mislocalization contributes to the increased proteasomal degradation of the mutant protein. While this cannot be entirely excluded, restoring the nuclear localization of the mutant ATOH7:R41_R48del protein by co-expressing E47:WT did not restore protein levels (Figure 3C). Furthermore, co-expression of E47:WT may in fact rather promote dimerization-mediated proteasomal degradation of ATOH7:WT, which is evidenced by an effect of MG-132 treatment in presence of E47:WT (Figure 3C) but not in its absence (Figure 3A).

### 3.3. The ATOH7 Basic Domain Plays a Role in Protein Dimerization

It is well documented that dimerization of bHLH proteins relies on the HLH domain, whereas the basic domain is important for DNA-binding [3,43]. Our findings suggest however that the basic domain may also influence protein interaction (Figure 4C,D) and are consistent with our previous observation that the mutant protein ATOH7:N46H may interfere with dimerization [29]. In previous work on characterizing the basic domain of the bHLH transcription factor myoblast determination protein 1 (MYOD1; OMIM: * 159970), it was reported that various amino acid exchanges within the basic domain affect the DNA-binding of heterodimers formed between MYOD1 and E12 [15,44]. Albeit most of these mutations did not impact heterodimerization with E12, substitutions in the MYOD1 basic regions BS2 and BS3, corresponding to ATOH7 Arg40–Arg42 and Arg49–Arg51, showed reduced dimerization [15].

### 3.4. Mutation of the ATOH7 Basic Domain Reduces Transcriptional Activation and Inhibits the Ability of Heterodimers to Regulate Transcription

The mutant ATOH7:R41_R48del protein exhibited a significant reduction in transcriptional activation, both in the absence and also in the presence of the dimerization partner E47, and was not affected by increased protein amounts through proteasomal inhibition (Figure 5C,D). This finding is consistent with our previous observation that ATOH7:N46H is a loss-of-function mutation [26,29]. Interestingly, both ATOH7:R41_R48del and N46H also provide evidence for decreasing the ability of E47 to activate transcription. It is likely that ATOH7 protein variants with a mutation in the basic domain act in analogy to negative E-proteins, also known as inhibitors of DNA-binding (Id-proteins), which are capable of heterodimerization with E47. Heterodimers subsequently possess reduced DNA-binding due to the lack of a DNA-binding domain [45]. This analogy would thus explain the reduced E47-mediated transcriptional activation in presence of the mutant ATOH7:R41_R48del protein (Figure 5C). In contrast, the previously published helix 1 mutations ATOH7:A59T and A59V displayed loss of transcriptional activation but did not affect the baseline activation levels induced by E47 [29]. This may prove to be an important distinction between basic and HLH domain mutations, which could potentially explain the large phenotypical differences and clinical variability in *ATOH7*-associated eye disorders.

The impact of the His46 residue on DNA-binding and thus transcriptional activation is also supported by previous work on determining the crystal structure of the NEUROD1-E47 dimer, which identifies the most important DNA-binding amino acids to correspond to the ATOH7 amino acids His46, Glu49, Arg50 and Arg52 [4]. Moreover, the loss of Arg40–Arg41 in the mutant ATOH7 protein may also have a significant impact on transcriptional activation in analogy to MYOD1, where mutation of the corresponding amino acid positions in the MYOD1 basic domain resulted in a strong reduction in transcriptional activation of a reporter gene [46].

### 3.5. The Basic Domain Mediates Various Functional Properties of the ATOH7 Protein

Given the findings in this work, the role of the ATOH7 basic domain can be summarized by five key functions; (1) intracellular localization and transport, as evidenced by mislocalization of the mutant ATOH7:R41_R48del protein and in silico prediction of a bipartite NLS; (2) protein stability, as the mutant ATOH7:R41_R48del protein shows a significantly increased proteasome-dependent turnover and reduced protein amounts; (3) protein heterodimerization; evidenced by decreased binding of the mutant ATOH7:R41_R48del protein to the putative heterodimerization partner E47 in living cells; (4) DNA-binding; as shown by reduced E-box-mediated enrichment of mutant ATOH7:R41_R48del protein in cell-free assays; and finally (5) transcriptional activation, as the mutant ATOH7:R41_R48del protein shows significantly reduced transcriptional activation and simultaneously a decrease in E47-induced transcriptional reporter activation.

### 3.6. Conclusions

The *ATOH7* mutation NM_145178.4:c.121_144del; p.(Arg41_Arg48del), previously described in a young patient suffering from NCRNA, leads to an in-frame deletion of eight amino acids in the basic domain of the transcription factor. Utilizing this disease-associated mutation as a basis for characterizing the role of the ATOH7 basic domain, we concluded that this domain is not only important in DNA-binding and mediating transcriptional activation, but extends its function to also regulate nuclear transport, protein stability, as well as protein heterodimerization. Besides the functional aspects of the ATOH7 basic domain, we also provide indications of extranuclear heterodimerization and synergistic nuclear import, which have not been previously reported for ATOH7 and were only sparsely described for bHLH transcription factors.

## 4. Materials and Methods

### 4.1. Bioinformatics Analysis

Protein sequences of atonal homologs of twelve different species were retrieved from the reference sequence (RefSeq) database at the national center for biotechnology information (NCBI; Bethesda, MD, USA; https://www.ncbi.nlm.nih.gov/refseq; accessed on 18 August 2020). MSA of the bHLH domains was performed using ClustalW2 (EMBL-EBI, Hinxton, UK; https://www.ebi.ac.uk/Tools/msa/clustalw2; accessed on 18 August 2020) [47]. Sequence alignment was carried out with the following reference sequences: NP_660161.1 (*Homo sapiens*), XP_521492.2 (*Pan troglodytes*), XP_032026345.1 (*Hylobates moloch*), NP_001163953.1 (*Rattus norvegicus*), NP_058560.1 (*Mus musculus*), XP_022273194.1 (*Canis lupus familiaris*), XP_001508483.2 (*Ornithorhynchus anatinus*), NP_989999.1 (*Gallus gallus*), NP_001079290.1 (*Xenopus laevis*), TWW78083.1 (*Takifugu flavidus*), NP_571707.1 (*Danio rerio*) and NP_731223.1 (*Drosophila melanogaster*).

### 4.2. Protein Modeling

Protein modeling of human ATOH7 (NP_660161) was generated by comparative protein structure modeling using the M4T server version 3.0 (http://manaslu.fiserlab.org/M4T; accessed on 1 October 2012) [48,49]. The bHLH crystal structure of the DNA-bound mouse heterodimer NEUROD1-E47 (protein data bank: 2ql2) was used as a template [4]. Figures were generated using the molecular visualization program PYMOL version 1.4.1 (Schrödinger, New York, NY, USA).

### 4.3. Expression Vectors

The coding sequence of *ATOH7* (RefSeq: NM_145178.4) was amplified from gDNA of a patient carrying the deletion in the basic domain and a healthy control by PCR using 3.0 U Pfu^®^ Polymerase (Promega, Madison, WI, USA), 1X Pfu Buffer, 0.16 mm dNTPs and 0.20 μM primers (ATOH7_CACC_for and ATOH7_STOP_rev (Table S2)) in 50 μL reaction volume. The amplified fragments were cloned into the expression vector pcDNA3.1/nV5-DEST (Thermo Fisher Scientific, Waltham, MA, USA), which expresses an N-terminal V5 tag under the control of a cytomegalovirus (CMV) promoter. Cloning was performed using the Gateway cloning system (Invitrogen, Carlsbad, CA, USA).

Constructs containing an N-terminal NLS from the SV-40 large T-antigen (Pro-Lys-Lys-Lys-Arg-Lys-Val) were generated by site-directed mutagenesis using the primers NLS_ATOH7_for and ATOH7_STOP_rev (Table S2). *TCF3(E47)* cDNA (RefSeq: NM_001136139.4) was synthesized *de novo* (OriGene, Rockville, MD, USA) and cloned into the pcDNA3.1/nV5-DEST expression vector, which was modified to express a C-terminal c-Myc tag. The E47:ΔNLS construct was generated by introducing c.472_522del; p.(Arg158_Arg174del) by site-directed mutagenesis using the primers TCF3E47:ΔNLS_for and TCF3E47:ΔNLS_rev (Table S2). All site-directed mutagenesis products were verified by Sanger sequencing and were subsequently re-cloned into the original backbone in order to avoid PCR-mediated vector mutations.

### 4.4. Cell Culture and Protein Assays

Maintenance and transfection of HEK293T, immunocytochemistry, WB, CHX chase assay, ELISA and dual luciferase assay are described in much detail in our previously published work [29].

### 4.5. Confocal Fluorescence Microscopy

Microscopy was performed on an SP8 Instrument (Leica Microsystems CMS GmbH, Wetzlar, Germany) using a 63 × HC PL APO CS2 oil immersion objective (NA 1.40). Overview images were acquired at 0.75 × zoom and at 4096 × 4096 px resolution (60 × 60 nm/px) and magnified orthogonal sections were acquired at 4.2 × zoom and at 512 × 512 px resolution (43 × 43 nm/px). Z-stacks were taken at 131 nm step size. Laser sources were set to 405, 488 and 633 nm, respectively. Images were acquired and exported using the Leica Application Suite X version 3.7.1 (Leica Microsystems CMS GmbH, Wetzlar, Germany) and were subsequently deconvoluted in Huygens Professional version 20.10.0p2 (Scientific Volume Imaging, Hilversum, The Netherlands). Orthogonal sections were prepared in Imaris x 64 version 9.7.2 (Bitplane AG, Zurich, Switzerland).

### 4.6. FLIM–FRET

The fluorescent protein mNeonGreen was chosen as the donor fluorophore due to its brightness, high photostability, fast maturation as well as a mono-exponential lifetime decay behavior in FLIM experiments [50,51]. As acceptor fluorophore, mRuby3 was chosen due to high brightness, photostability, extinction coefficient and quantum yield, as well as good overlap of its absorbance spectrum with the emission spectrum of mNeonGreen (Figure S8) [52]. The donor expression construct ATOH7-mNeonGreen, the acceptor construct vector E47-mRuby3 and variants thereof (Table S1) were synthesized and cloned into a pcDNA3.1 vector (BioCat, Heidelberg, Germany).

HEK293T cells (Sigma-Aldrich, St. Louis, MO, USA) were seeded at a density of 1.0 × 10^5^ viable cells per well onto poly-L-lysine pretreated µ-Slide 8-Well Glass Bottom Cell Culture Chambers (Ibidi GmbH, Gräfelfing, Germany). On the following day, a total of 600 ng plasmid DNA was transfected using branched polyethylenimine (Sigma-Aldrich, St. Louis, MO, USA) in a 3:1 ratio with DNA. Cells were incubated in 36.5 °C and 5.0% CO_2_ for three days following transfection with daily medium changes and were treated with 20 mM MG-132 (Selleck Chemicals Llc, Houston TX, USA) 4 h before imaging. Immediately before FLIM, medium was changed to an imaging buffer consisting of serum-free and phenol-free Dulbecco’s Modified Eagle’s Medium (DMEM; Thermo Fisher Scientific, Waltham, MA, USA) supplemented with 1.0 mM sodium pyruvate and 4.0 mM L-glutamine (Thermo Fisher Scientific, Waltham, MA, USA).

FLIM measurements were performed on an SP8 Fast Lifetime Contrast FALCON Instrument (Leica Microsystems CMS GmbH, Wetzlar, Germany) using a live cell incubation system at 36.5 °C in 5.0% CO_2_ and 60% humidity. Imaging was performed using a 63 × HC PL APO CS2 glycerol immersion objective (NA 1.30), a supercontinuum laser source with the pulse picker at 40 MHz and hybrid photo detectors running in photon counting mode. Donor FLIM acquisition was performed at 514 nm excitation and detection was performed using three aggregated detectors set to 519–555 nm, whereas acceptor acquisition was performed at 561 nm excitation and detection was performed using a single detector set to 650–700 nm. All image acquisitions and calculations were performed in the Leica Application Suite X FLIM/FCS version 3.5.6 (Leica Microsystems CMS GmbH, Wetzlar, Germany).

For each condition, three independent experiments were performed with three image acquisitions per experiment. A total of at least 38 individual cells were measured per condition. The donor lifetime was fitted by mono-exponential reconvolution using a simulated instrument response function. Subsequently, FRET calculations were performed using a mono-exponential donor model. FLIM measurements were averaged for each region of interest. The FLIM–FRET efficiency *E* was calculated according to Equation (1), the apparent FRET efficiency *E_App_* was calculated according to Equation (2), the donor–acceptor binding ratio *B*_%_ was calculated according to Equation (3) and the donor–acceptor distance *r* was derived from Equation (4), where a Förster radius of *R*_0_ = 6.417 nm was used (FPbase FRET Calculator, Harvard Medical School, Boston, MA, USA; https://www.fpbase.org/fret) [46]. All data are presented as boxplots showing medians ± quartiles and 10–90 percentile whiskers. Significance was tested using the Kruskal–Wallis nonparametric test corrected for multiple comparisons using Dunn’s test, with a significance and confidence level of *p* < 0.05.

(1) $$E=\frac{\tau_{UD}-\tau_{QD}}{\tau_{UD}}$$

(2) $$E_{App}=\frac{I_{QD}}{\sum I}*E$$

(3) $$B_{\%}=\frac{A_{QD}}{A_{UQ}+A_{QD}}$$

(4) $$E=\frac{R_{0}{}^{6}}{R_{0}{}^{6}+r^{6}}$$

Equations used in FLIM–FRET calculations. *A*, amplitude; *B*_%_, donor–acceptor binding ratio; *E*, FLIM–FRET efficiency; *I* intensity; *τ*, fluorescence lifetime; *QD*, quenched donor fraction; *R*_0_, Förster radius; *r*, donor–acceptor distance; *UD*, unquenched donor fraction.

### 4.7. Statistical Analysis

Calculations and figures were made in GraphPad PRISM v6.07 (GraphPad Software Inc., San Diego, CA, USA). Unless specified otherwise, all data are shown as means ± SD and testing of significance was performed by either one-way ANOVA for column statistics or two-way ANOVA with correction for multiple comparisons using Holm–Šidák for grouped analysis.

## Figures and Tables

**Table 1:** List of published *ATOH7* variants and associated phenotypes. Note that the heterozygous variants do not show a clear genotype–phenotype relationship. Transcription activity refers to whether the protein induces transcriptional activation of a reporter gene in luciferase assays. *ATOH7*, atonal bHLH transcription factor 7; dbSNP, single-nucleotide polymorphism database; EVR, exudative vitreoretinopathy; FVH, foveal hypoplasia; HGMD, human gene mutation database; HMZ, homozygous; HTZ, heterozygous; LOF, loss of function; NCRNA, nonsyndromic congenital retinal nonattachment; ONH, optic nerve hypoplasia; PHPVAR, autosomal recessive persistent hyperplastic primary vitreous; POAG, primary open-angle glaucoma; UTR, untranslated region.

Identifier	DNA/Protein Variant	Region	Phenotype(s)	Zygosity	Segregation	Transcription Activity	Published
CM1213359 ^1^	c.139G>A; p.(Ala47Thr)	Basic	ONH	HTZ	–	Reduced	2010 [26,30]
C0027672 ^2^	c.193A>G; p.(Arg65Gly)	Helix 1	ONH	HTZ	–	Normal	2010 [26,30]
CG115298 ^1^	c.-22208_-15686del	Enhancer	NCRNA, ONH	HMZ	Yes	–	2011 [24]
rs1900004 ^3^	c.-9021C>T	5′-UTR	POAG	–	–	–	2011 [31]
CM121097 ^1^	c.53delC; p.(Pro18ArgfsTer69)	Coding *	NCRNA, ONH	HMZ	Yes	–	2012 [25]
CM121094 ^1^	c.146A>T; p.(Glu49Val)	Basic	NCRNA, ONH	HMZ	Yes	–	2012 [25]
CM126904 ^1^	c.136A>C; p.(His46Asn)	Basic	PHPVAR	HMZ	Yes	LOF	2012 [26]
CG1613845 ^1^	c.121_144del; p.(Arg41_Arg48del)	Basic	NCRNA EVR	HMZ HTZ	Yes No	–	2016 [27]
CM1613857 ^1^	c.400G>T; p.(Glu134 *)	Helix 2	EVR	HTZ	No	–	2017 [27]
CM172971 ^1^	c.125G>C; p.(Arg42Pro)	Basic	NCRNA	HMZ	Yes	–	2017 [28]
CM204439 ^1^ CM204440 ^1^	c.175G>A; p.(Ala59Thr) c.176C>T; p.(Ala59Val)	Helix 1	ONH, FVH	Compound HTZ	Yes	LOF	2020 [29]

^1^ HGMD (Qiagen, Hilden, Germany; http://www.hgmd.org; accessed on 15 March 2021). ^2^ MedGen (NCBI, Bethesda, MD, USA; https://www.ncbi.nlm.nih.gov/medgen; accessed on 15 March 2021). ^3^ dbSNP (NCBI, Bethesda, MD, USA; https://www.ncbi.nlm.nih.gov/snp; accessed on 15 March 2021). * Upstream of the bHLH motif.

## Data Availability

The data presented in this study are available on request from the corresponding author.

## Supplementary Materials

The following supporting information can be downloaded at: https://www.mdpi.com/article/10.3390/ijms23031053/s1.
