# Supplementary material for: Functional Characterization of an In-Frame Deletion in the Basic Domain of the Retinal Transcription Factor ATOH7
Source: Int J Mol Sci. 2022 Jan 19;23(3):1053. doi: 10.3390/ijms23031053 (PMC8834682; doi:10.3390/ijms23031053)
Supplement: Supplementary file 1 [file ijms-23-01053-s001.zip › ijms-1529878-supplementary.pdf]

Electronic Supplementary Material

Supplementary Figures

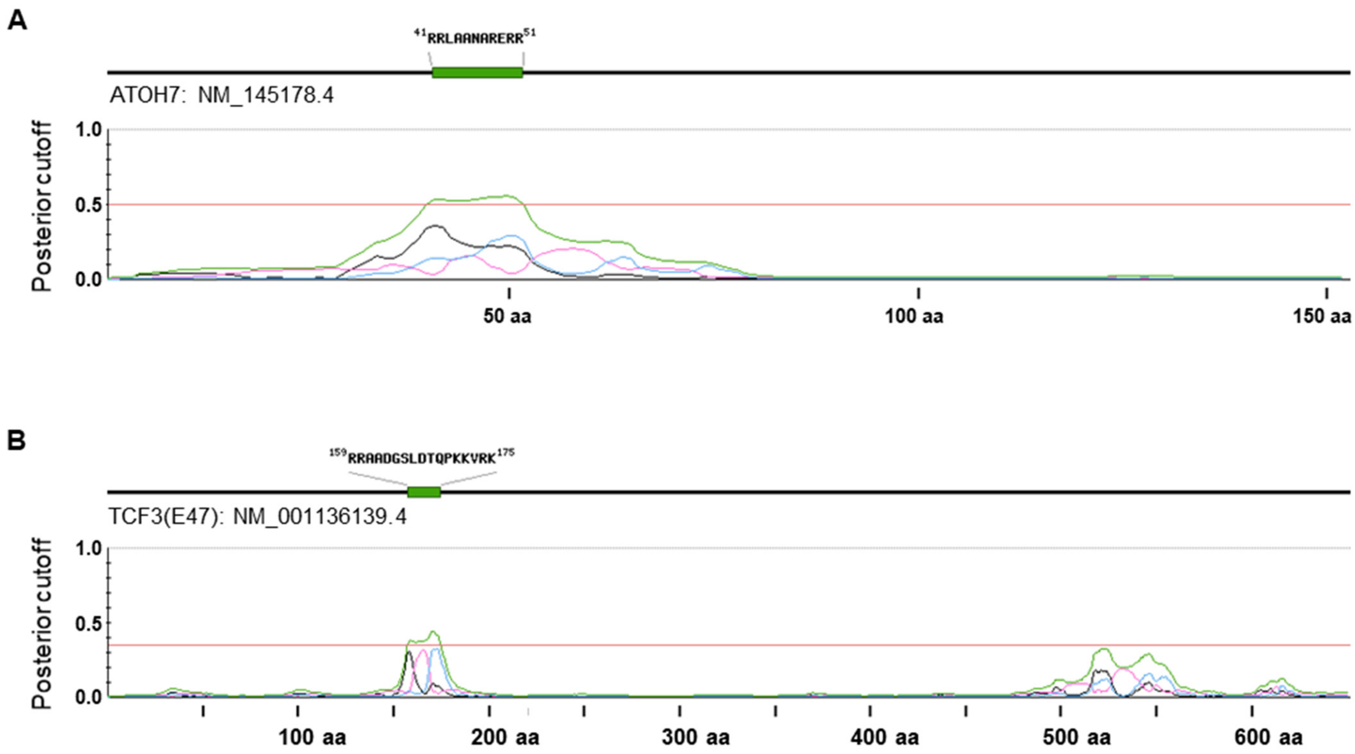

Figure S1. Putative bipartite NLS in ATOH7 and E47 predicted by NLStradamus. The predictions were based on a static four-state HMM with a set posterior cut-off. The predicted NLS region is marked in green. (**A**) Predicted NLS in ATOH7 (posterior cut-off: 0.50). (**B**) Predicted NLS in E47 (posterior cut-off: 0.35). aa, amino acid; ATOH7, atonal bHLH transcription factor 7; HMM, hidden Markov model; NLS, nuclear localization signal; E47, transcription factor 3 isoform E47.

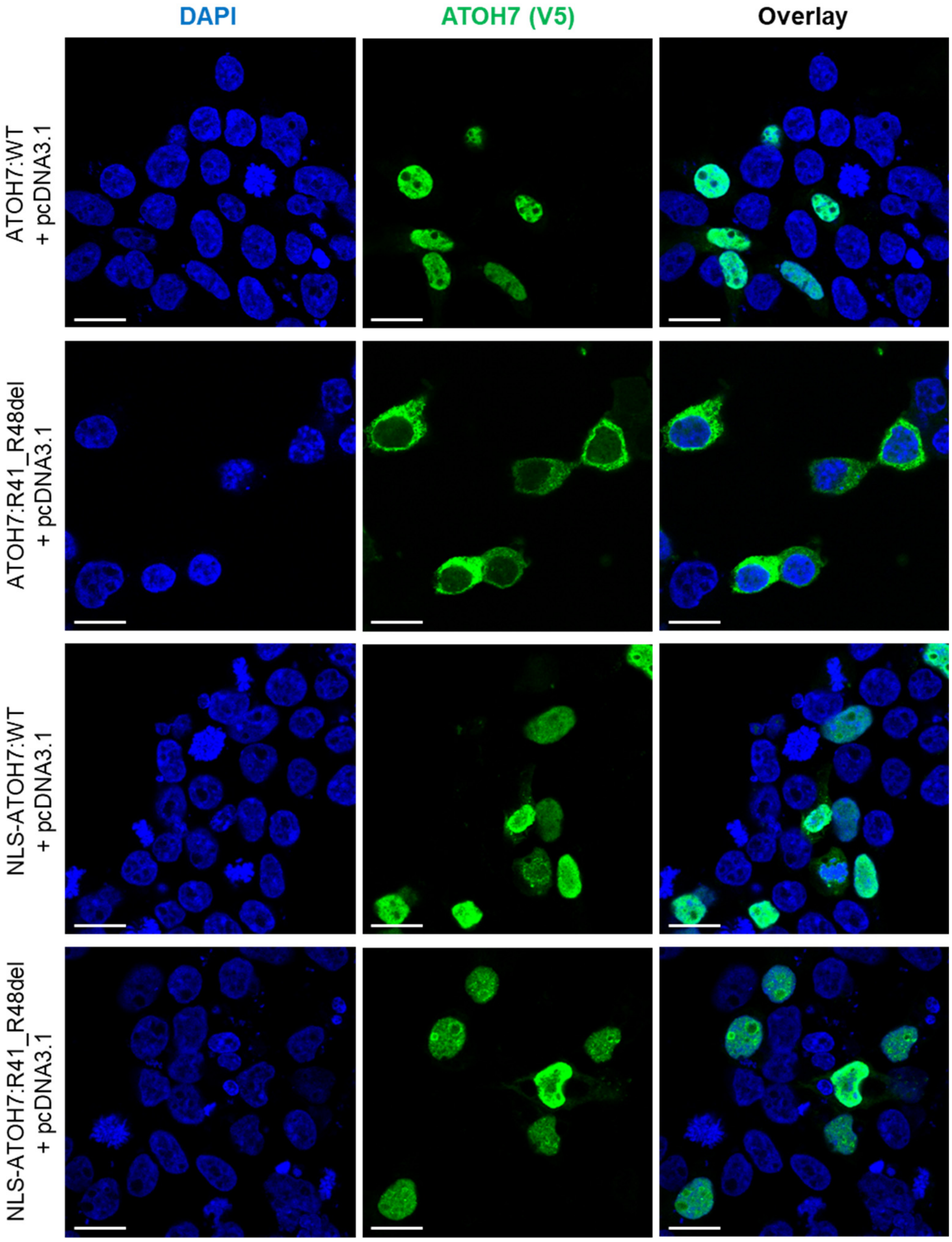

Figure S2. *Continued on next page.*

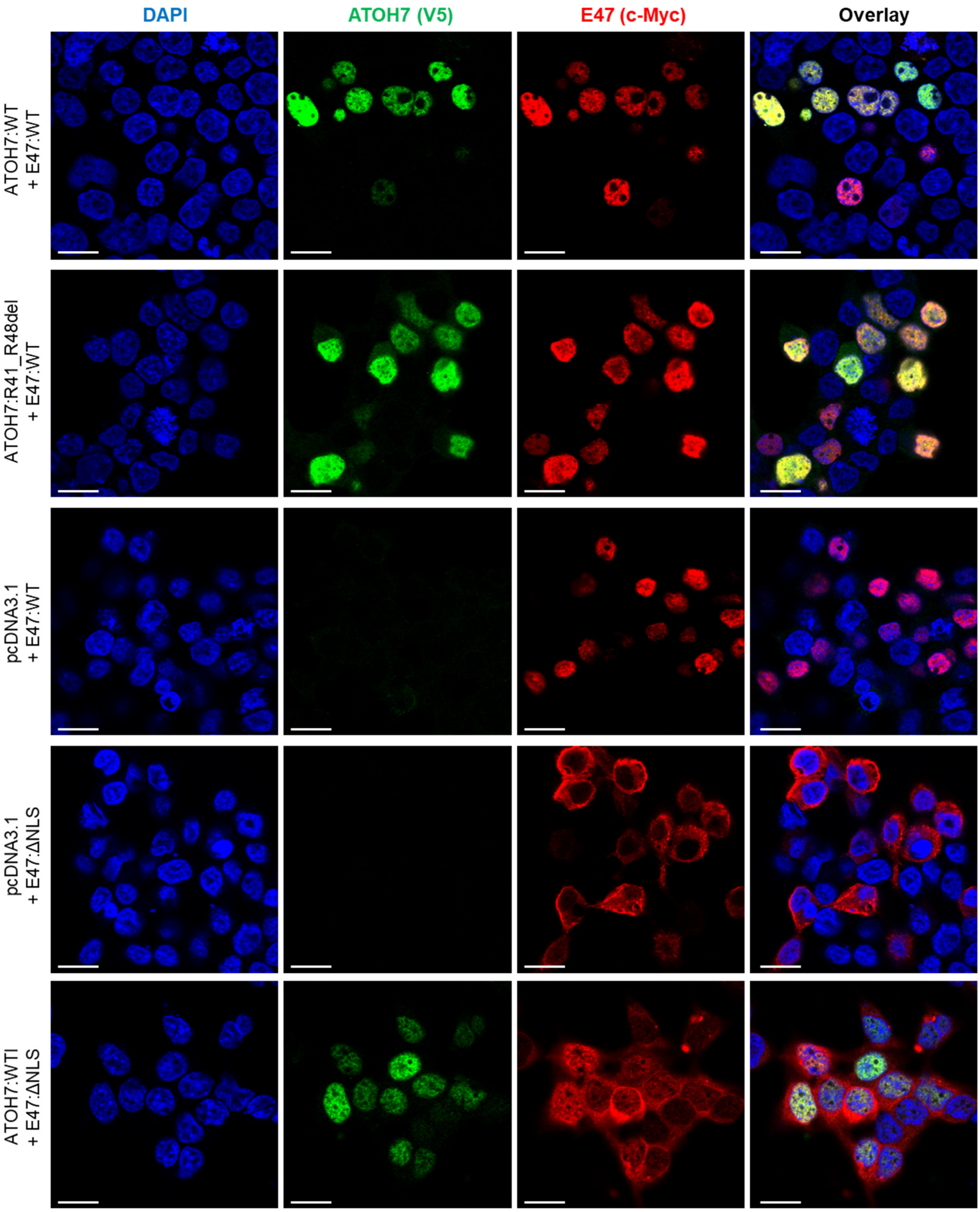

Figure S2. Supplementary confocal images. Transgenic V5-tagged ATOH7 (green) and c-Myc-tagged E47 (red) proteins were expressed in HEK293T cells without treatment with the protease inhibitor MG-132. Nuclei are stained by DAPI (blue). Scale bars represent 15 µm. ATOH7, atonal bHLH transcription factor 7; DAPI; 4′,6-diamidino-2-phenylindole; E47, transcription factor 3 isoform E47; HEK293T, human embryonic kidney 293T; NLS, nuclear localization signal; WT, wildtype.

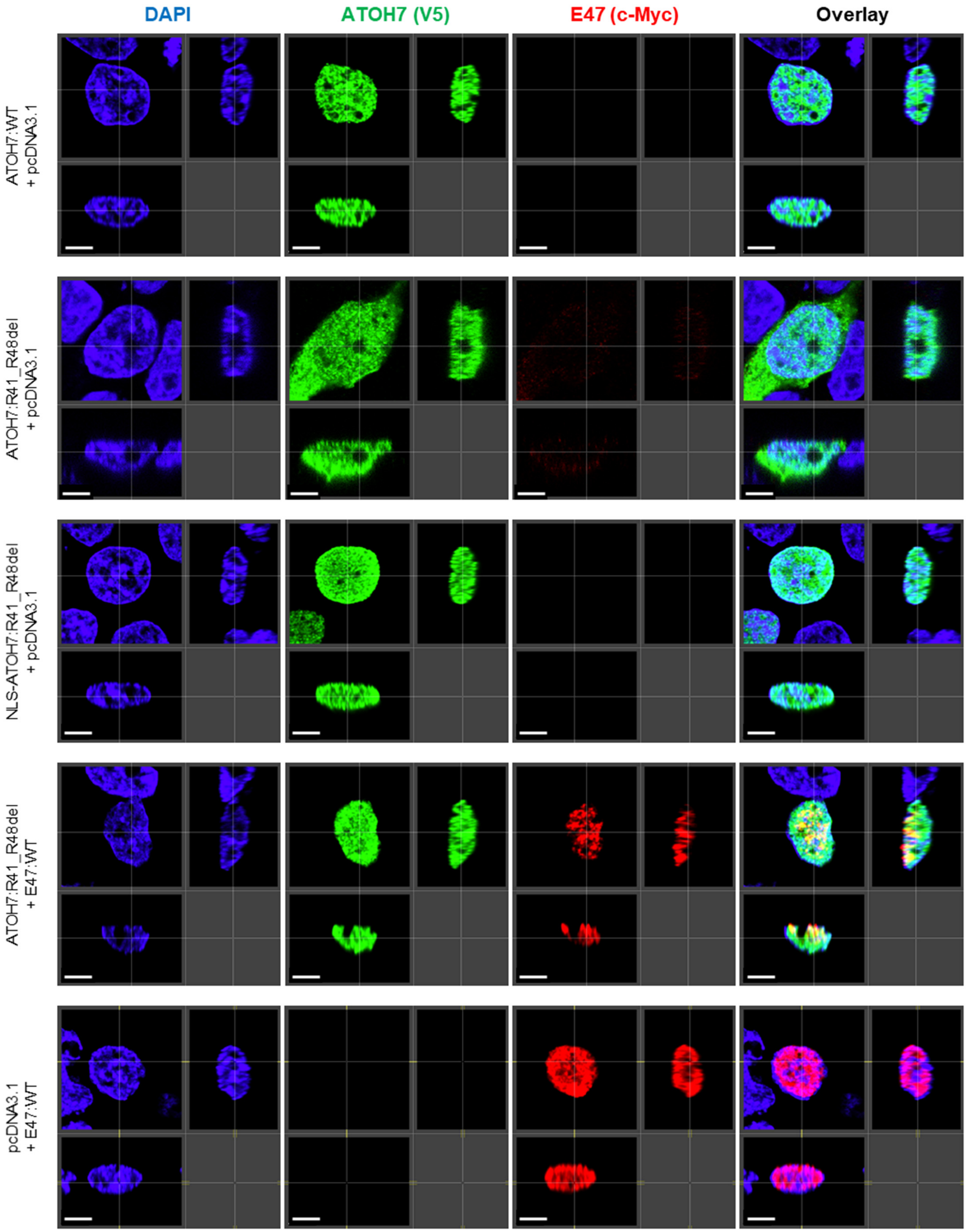

Figure S3. *Continued on next page.*

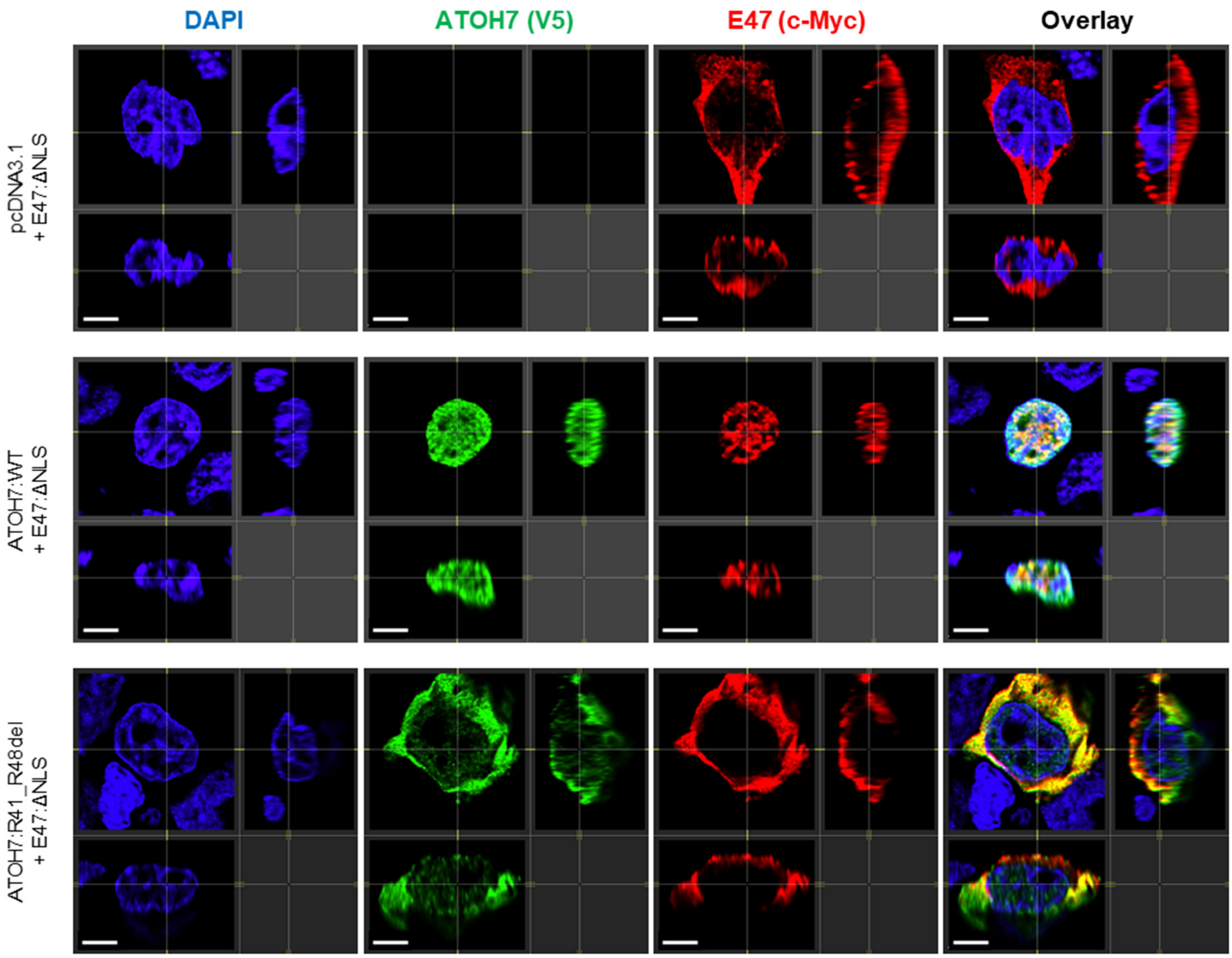

Figure S3. Supplementary orthogonal projections. The images represent separate channels of magnified orthogonal projections in Figure 2, depicting the subcellular localization of V5-tagged ATOH7 (green) and c-Myc-tagged E47 (red) proteins expressed in HEK293T cells after 4h of proteasomal inhibition by MG-132. Nuclei are stained by DAPI (blue). Scale bars represent 5 µm. ATOH7, atonal bHLH transcription factor 7; DAPI; 4′,6-diamidino-2-phenylindole; E47, transcription factor 3 isoform E47; HEK293T, human embryonic kidney 293T; NLS, nuclear localization signal; WT, wildtype.

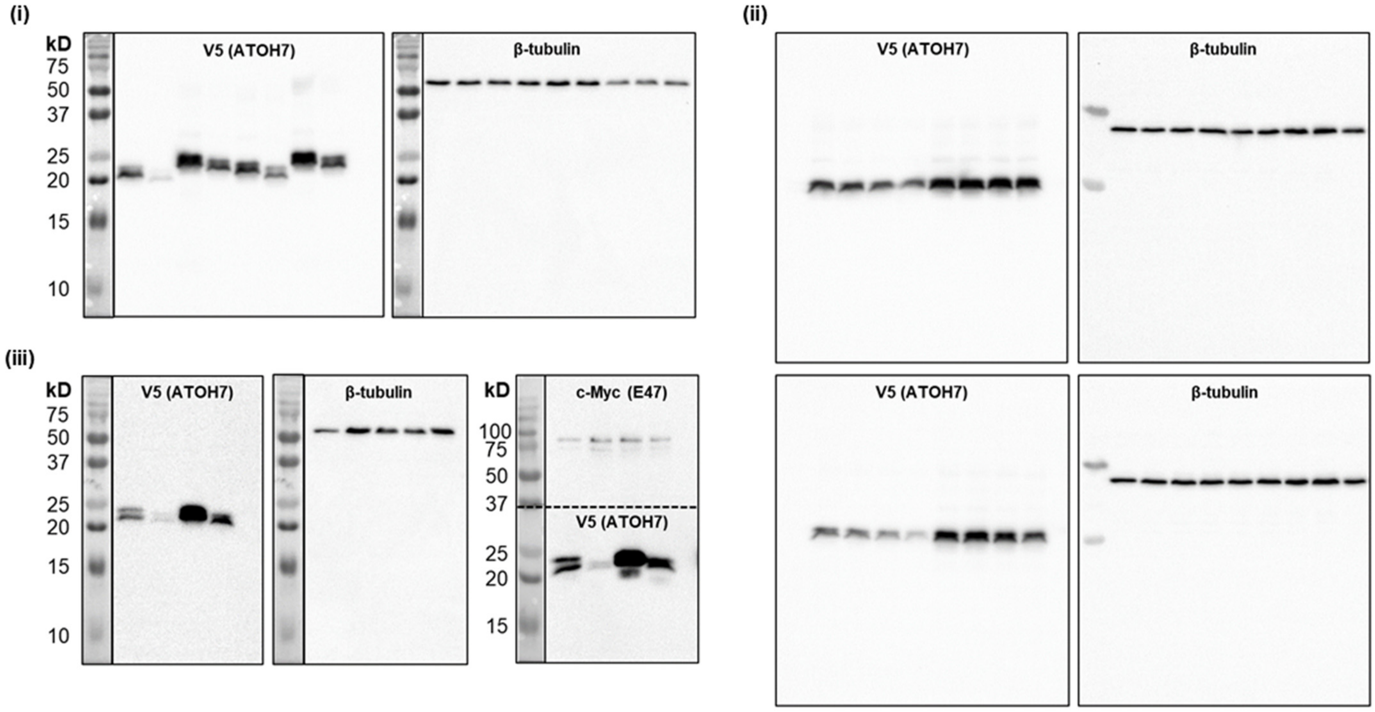

Figure S4. Representative SDS-PAGE blots corresponding to Figure 3D (i-iii). ATOH7, atonal bHLH transcription factor 7; E47, transcription factor 3 isoform E47; SDS-PAGE; sodium dodecyl sulfate polyacrylamide gel electrophoresis.

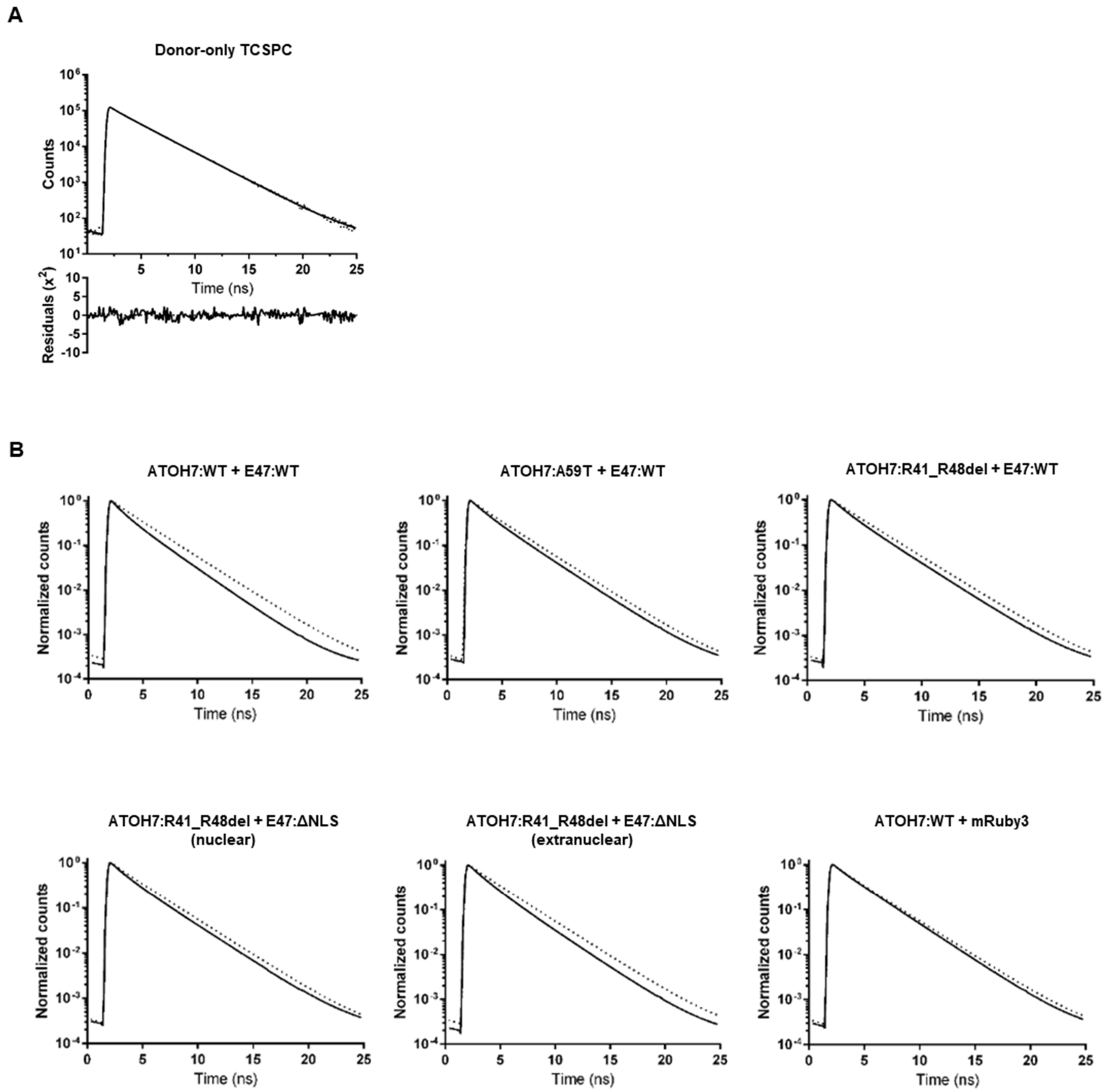

Figure S5. Examples of donor TCSPC graphs acquired during FLIM-FRET. (**A**) Donor-only control consisting of mono-expressed mNeonGreen-linked ATOH7 WT. (**B**) TCSPC graphs of mNeonGreen-linked ATOH7 donors when co-expressed with mRuby3-linked E47 acceptors. Each curve was normalized to the highest count and overlaid with donor-only control (dotted line). ATOH7, atonal bHLH transcription factor 7; E47, transcription factor 3 isoform E47; FLIM, fluorescence lifetime imaging; FRET, Förster resonance energy transfer; NLS, nuclear localization signal; TCSPC, time-correlated single photon counting; WT, wildtype.

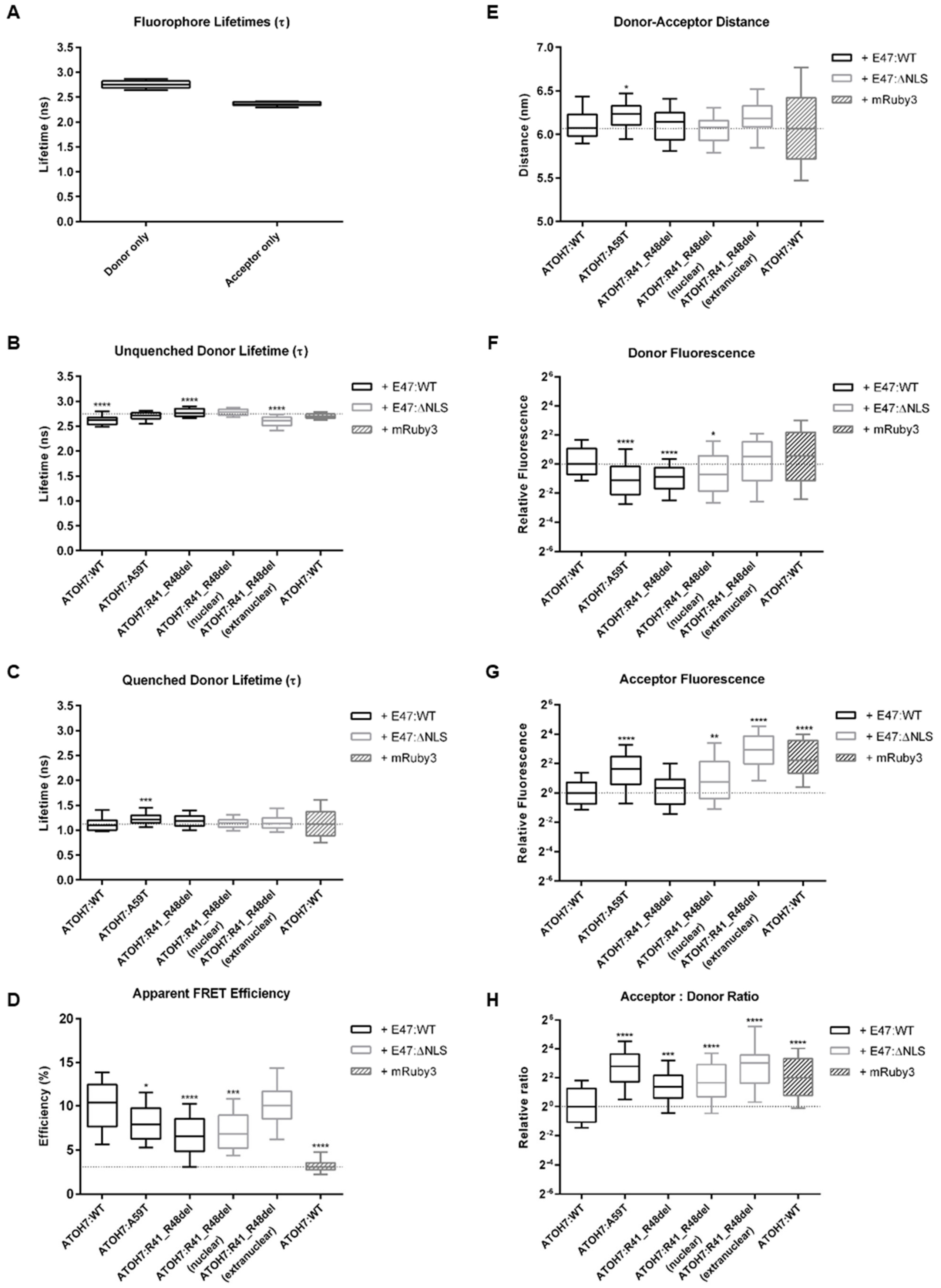

Figure S6. Protein interaction of ATOH7 and E47 in live cells acquired through FLIM-FRET. HEK293T cells were transfected with N-terminal mNeonGreen-linked ATOH7 donor and C-terminal Ruby3-linked E47 acceptor. Boxplots represent medians ± quartiles and

10-90 percentile whiskers of three independent experiments. *, **, ***, **** represent p-values of <0.05, $<10^{-2}$, $<10^{-3}$ and $<10^{-4}$, respectively. (**A**) Donor and acceptor fluorophore lifetimes. (**B**) UD lifetimes. Statistical significance was calculated relative the donor-only lifetime (dotted line). (**C-E**) QD lifetimes, apparent FRET efficiencies and donor-acceptor distances. Statistical significance was calculated relative ATOH7:WT interacting with E47:WT. The dotted lines indicate the corresponding value for the negative control, which consists of the ATOH7:WT donor interacting with the free mRuby3 acceptor. (**F**) Donor fluorescence shown as fold-change relative ATOH7:WT (dotted line). (**G**) Acceptor fluorescence shown as fold-change relative ATOH7:WT (dotted line). (**H**) A:D ratio shown as fold-change relative ATOH7:WT (dotted line). A:D ratio, acceptor-donor ratio; ATOH7, atonal bHLH transcription factor 7; E47, transcription factor 3 isoform E47; HEK293T, human embryonic kidney 293T; FLIM, fluorescence lifetime imaging; FRET, Förster resonance energy transfer; NLS, nuclear localization signal; UD, unquenched donor; QD, quenched donor; WT, wildtype.

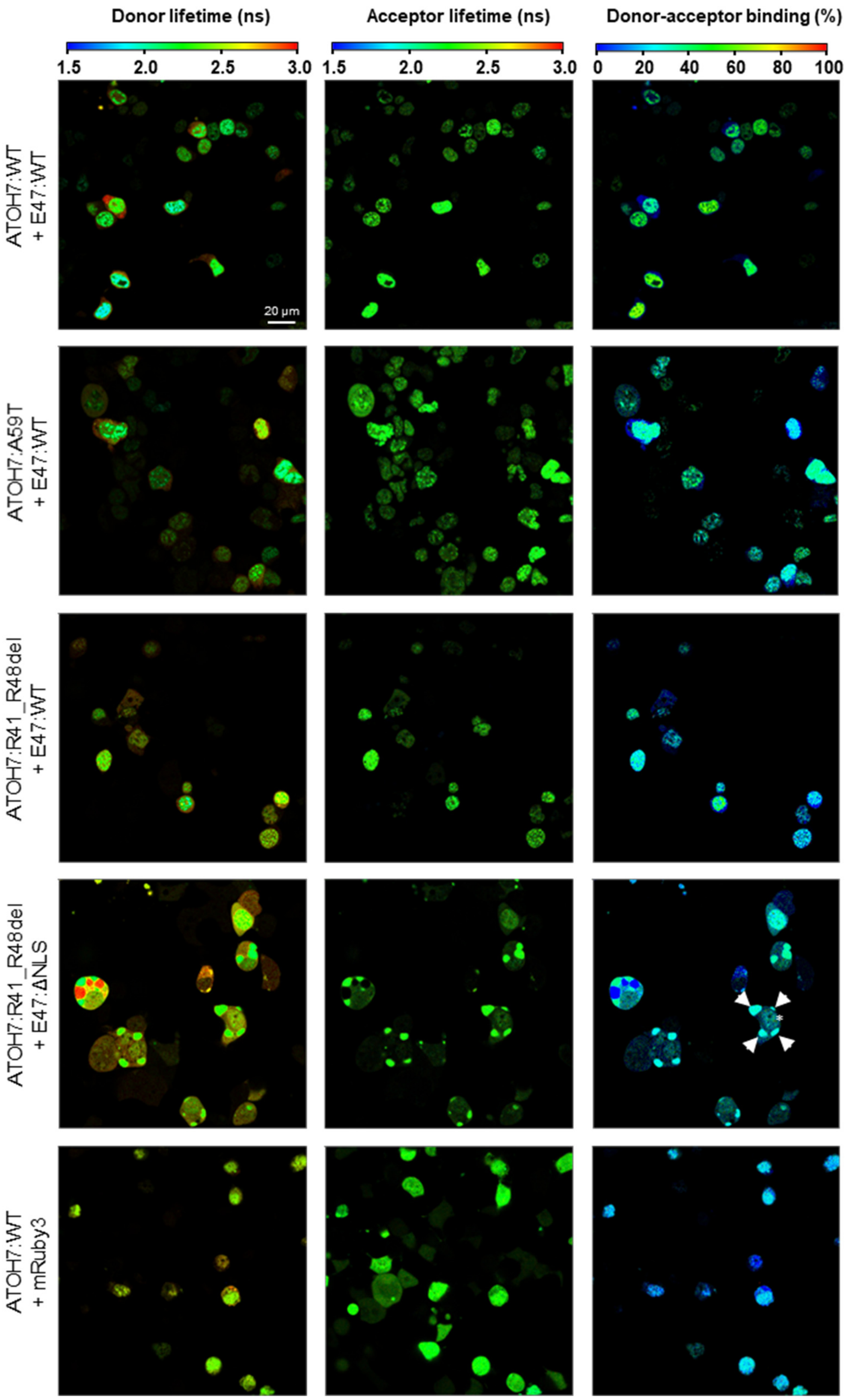

Figure S7. Example images of HEK293T cells visualized by FLIM-FRET. Heatmaps represent mNeonGreen-linked ATOH7 donor lifetimes, mRuby3-linked E47 acceptor lifetimes and fraction of donor-acceptor binding, respectively. Asterisk (*) marks the nucleus and arrows point to extranuclear areas. ATOH7, atonal bHLH transcription factor 7; E47, transcription factor 3 isoform E47; HEK293T, human embryonic kidney 293T; FLIM, fluorescence lifetime imaging; FRET, Förster resonance energy transfer; NLS, nuclear localization signal; WT, wildtype.

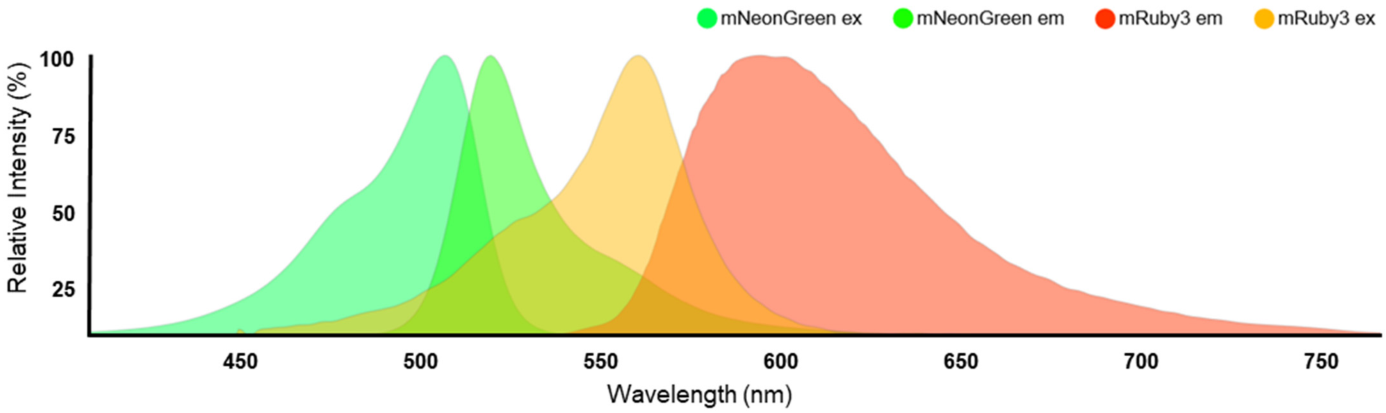

Figure S8. Excitation and emission spectra of mNeonGreen and mRuby3. The emission spectrum of mNeonGreen overlaps with the absorption spectrum of mRuby3 to a high degree, thus making these fluorophores a suitable donor-acceptor FRET pair. em, emission; ex, excitation; FRET, Förster resonance energy transfer.

Supplementary Tables

Table S1. FLIM-FRET expression constructs. FLIM, fluorescence lifetime imaging, FRET, Förster resonance energy transfer.

Construct	Sequence
**mNeonGreen**	1 ATGGTGAGCA AGGGCGAGGA GGACAACATG GCCAGCCTGC CCGCCACCCA CGAGCTGCAC
	61 ATCTTCGGCA GCATCAACGG CGTGGACTTC GACATGGTGG GCCAGGGCAC CGGCAACCCC
	121 AACGACGGCT ACGAGGAGCT GAACCTGAAG AGCACCAAGG GCGACCTGCA GTTCAGCCCC
	181 TGGATCCTGG TGCCCCACAT CGGCTACGGC TTCCACCAGT ACCTGCCCTA CCCCGACGGC
	241 ATGAGCCCCT TCCAGGCCGC CATGGTGGAC GGCAGCGGCT ACCAGGTGCA CAGAACCATG
	301 CAGTTCGAGG ACGGCGCCAG CCTGACCGTG AACTACAGAT ACACCTACGA GGGCAGCCAC
	361 ATCAAGGGCG AGGCCCAGGT GAAGGGCACC GGCTTCCCCG CCGACGGCCC CGTGATGACC
	421 AACAGCCTGA CCGCCGCCGA CTGGTGCAGA AGCAAGAAGA CCTACCCCAA CGACAAGACC
	481 ATCATCAGCA CCTTCAAGTG GAGCTACACC ACCGGCAACG GCAAGAGATA CAGAAGCACC
	541 GCCAGAACCA CCTACACCTT CGCCAAGCCC ATGGCCGCCA ACTACCTGAA GAACCAGCCC
	601 ATGTACGTGT TCAGAAAGAC CGAGCTGAAG CACAGCAAGA CCGAGCTGAA CTTCAAGGAG
	661 TGGCAGAAGG CCTTCACCGA CGTGATGGGC ATGGACGAGC TGTACAAGTA G
**mRuby3**	1 ATGGTGAGCA AGGGCGAGGA GCTGATCAAG GAGAACATGA GAATGAAGGT GGTGATGGAG
	61 GGCAGCGTGA ACGGCCACCA GTTCAAGTGC ACCGGCGAGG GCGAGGGCAG ACCCTACGAG
	121 GGCGTGCAGA CCATGAGAAT CAAGGTGATC GAGGGCGGCC CCCTGCCCTT CGCCTTCGAC
	181 ATCCTGGCCA CCAGCTTCAT GTACGGCAGC AGAACCTTCA TCAAGTACCC CGCCGACATC
	241 CCCGACTTCT TCAAGCAGAG CTTCCCCGAG GGCTTCACCT GGGAGAGAGT GACCAGATAC
	301 GAGGACGGCG GCGTGGTGAC CGTGACCCAG GACACCAGCC TGGAGGACGG CGAGCTGGTG
	361 TACAACGTGA AGGTGAGAGG CGTGAACTTC CCCAGCAACG GCCCCGTGAT GCAGAAGAAG
	421 ACCAAGGGCT GGGAGCCCAA CACCGAGATG ATGTACCCCG CCGACGGCGG CCTGAGAGGC
	481 TACACCGACA TCGCCCTGAA GGTGGACGGC GGCGGCCACC TGCACTGCAA CTTCGTGACC
	541 ACCTACAGAA GCAAGAAGAC CGTGGGCAAC ATCAAGATGC CCGGCGTGCA CGCCGTGGAC
	601 CACAGACTGG AGAGAATCGA GGAGAGCGAC AACGAGACCT ACGTGGTGCA GAGAGAGGTG
	661 GCCGTGGCCA AGTACAGCAA CCTGGGCGGC GGCATGGACG AGCTGTACAA GTAG
**ATOH7:WT**	1 ATGAAGTCCT GCAAGCCCAG CGGCCCGCCG GCGGGAGCGC GCGTTGCACC CCCGTGCGCG
	61 GGCGGCACCG AGTGCGCGGG CACGTGCGCC GGGGCCGGGC GGCTGGAGAG CGCGGCGCGC
	121 AGGCGCCTGG CGGCCAACGC GCGCGAGCGC CGCCGCATGC AGGGGCTCAA CACTGCCTTC
	181 GACCGCTTAC GCAGGGTGGT TCCCCAGTGG GGCCAGGATA AAAAGCTGTC CAAGTACGAG
	241 ACCCTGCAGA TGGCCCTGAG CTACATCATG GCTCTGACCC GGATCCTGGC CGAGGCCGAG
	301 CGATTCGGCT CGGAGCGGGA CTGGGTGGGT CTCCACTGTG AGCACTTCGG CCGCGACCAC
	361 TACCTCCCGT TCCCGGGCGC GAAGCTGCCG GGCGAGAGCG AGCTGTACAG CCAGAGACTC
	421 TTCGGCTTCC AGCCCGAGCC CTTCCAGATG GCCACC
**ATOH7:R41_R48del**	1 ATGAAGTCCT GCAAGCCCAG CGGCCCGCCG GCGGGAGCGC GCGTTGCACC CCCGTGCGCG
	61 GGCGGCACCG AGTGCGCGGG CACGTGCGCC GGGGCCGGGC GGCTGGAGAG CGCGGCGCGC
	121 GAGCGCCGCC GCATGCAGGG GCTCAACACT GCCTTCGACC GCTTACGCAG GGTGGTTCCC
	181 CAGTGGGGCC AGGATAAAAA GCTGTCCAAG TACGAGACCC TGCAGATGGC CCTGAGCTAC
	241 ATCATGGCTC TGACCCGGAT CCTGGCCGAG GCCGAGCGAT TCGGCTCGGA GCGGGACTGG
	301 GTGGGTCTCC ACTGTGAGCA CTTCGGCCGC GACCACTACC TCCCGTTCCC GGGCGCGAAG
	361 CTGCCGGGCG AGAGCGAGCT GTACAGCCAG AGACTCTTCG GCTTCCAGCC CGAGCCCTTC
	421 CAGATGGCCA CC
**ATOH7:A59T**	1 ATGAAGTCCT GCAAGCCCAG CGGCCCGCCG GCGGGAGCGC GCGTTGCACC CCCGTGCGCG
	61 GGCGGCACCG AGTGCGCGGG CACGTGCGCC GGGGCCGGGC GGCTGGAGAG CGCGGCGCGC
	121 AGGCGCCTGG CGGCCAACGC GCGCGAGCGC CGCCGCATGC AGGGGCTCAA CACTACCTTC
	181 GACCGCTTAC GCAGGGTGGT TCCCCAGTGG GGCCAGGATA AAAAGCTGTC CAAGTACGAG

Table S1. *Cont.*

	241 ACCCTGCAGA TGGCCCTGAG CTACATCATG GCTCTGACCC GGATCCTGGC CGAGGCCGAG<br>301 CGATTCGGCT CGGAGCGGGA CTGGGTGGGT CTCCACTGTG AGCACTTCGG CCGCGACCAC<br>361 TACCTCCCGT TCCCGGGCGC GAAGCTGCCG GGCGAGAGCG AGCTGTACAG CCAGAGACTC<br>421 TTCGGCTTCC AGCCCGAGCC CTTCCAGATG GCCACC
**E47:WT**	1 ATGAACCAGC CGCAGAGGAT GGCGCCTGTG GGCACAGACA AGGAGCTCAG TGACCTCCTG<br>61 GACTTCAGCA TGATGTTCCC GCTGCCTGTC ACCAACGGGA AGGGCCGGCC CGCCTCCCTG<br>121 GCCGGGGCGC AGTTCGGAGG TTCAGGTCTT GAGGACCGGC CCAGCTCAGG CTCCTGGGGC<br>181 AGCGGCGACC AGAGCAGCTC CTCCTTTGAC CCCAGCCGGA CCTTCAGCGA GGGCACCCAC<br>241 TTCACTGAGT CGCACAGCAG CCTCTCTTCA TCCACATTCC TGGGACCGGG ACTCGGAGGC<br>301 AAGAGCGGTG AGCGGGGCGC CTATGCCTCC TTCGGGAGAG ACGCAGGCGT GGGCGGCCTG<br>361 ACTCAGGCTG GCTTCCTGTC AGGCGAGCTG GCCCTCAACA GCCCCGGGCC CCTGTCCCCT<br>421 TCGGGCATGA AGGGGACCTC CCAGTACTAC CCCTCCTACT CCGGCAGCTC CCGGCGGAGA<br>481 GCGGCAGACG GCAGCCTAGA CACGCAGCCC AAGAAGGTCC GGAAGGTCCC GCCGGGTCTT<br>541 CCATCCTCGG TGTACCCACC CAGCTCAGGT GAGGACTACG GCAGGGATGC CACCGCCTAC<br>601 CCGTCCGCCA AGACCCCCAG CAGCACCTAT CCCGCCCCCT TCTACGTGGC AGATGGCAGC<br>661 CTGCACCCCT CAGCCGAGCT CTGGAGTCCC CCGGGCCAGG CGGGCTTCGG GCCCATGCTG<br>721 GGTGGGGGCT CATCCCCGCT GCCCCTCCCG CCCGGTAGCG GCCCGGTGGG CAGCAGTGGA<br>781 AGCAGCAGCA CGTTTGGTGG CCTGCACCAG CACGAGCGTA TGGGCTACCA GCTGCATGGA<br>841 GCAGAGGTGA ACGGTGGGCT CCCATCTGCA TCCTCCTTCT CCTCAGCCCC CGGAGCCACG<br>901 TACGGCGGCG TCTCCAGCCA CACGCCGCCT GTCAGCGGGG CCGACAGCCT CCTGGGCTCC<br>961 CGAGGGACCA CAGCTGGCAG CTCCGGGGAT GCCCTCGGCA AAGCACTGGC CTCGATCTAC<br>1021 TCCCCGGATC ACTCAAGCAA TAACTTCTCG TCCAGCCCTT CTACCCCCGT GGGCTCCCCC<br>1081 CAGGGCCTGG CAGGAACGTC ACAGTGGCCT CGAGCAGGAG CCCCCGGTGC CTTATCGCCC<br>1141 AGCTACGACG GGGGTCTCCA CGGCCTGCAG AGTAAGATAG AAGACCACCT GGACGAGGCC<br>1201 ATCCACGTGC TCCGCAGCCA CGCCGTGGGC ACAGCCGGTG ACATGCACAC GCTGCTGCCT<br>1261 GGCCACGGGG CGCTGGCCTC AGGTTTCACC GGCCCCATGT CACTGGGCGG GCGGCACGCA<br>1321 GGCCTGGTTG GAGGCAGCCA CCCCGAGGAC GGCCTCGCAG GCAGCACCAG CCTCATGCAC<br>1381 AACCACGCGG CCCTCCCCAG CCAGCCAGGC ACCCTCCCTG ACCTGTCTCG GCCTCCCGAC<br>1441 TCCTACAGTG GGCTAGGGCG AGCAGGTGCC ACGGCGGCCG CCAGCGAGAT CAAGCGGGAG<br>1501 GAGAAGGAGG ACGAGGAGAA CACGTCAGCG GCTGACCACT CGGAGGAGGA GAAGAAGGAG<br>1561 CTGAAGGCCC CCCGGGCCCG GACCAGCAGT ACGGACGAGG TGCTGTCCCT GGAGGAGAAA<br>1621 GACCTGAGGG ACCGGGAGAG GCGCATGGCC AATAACGCGC GGGAGCGGGT GCGCGTGCGG<br>1681 GATATTAACG AGGCCTTCCG GGAGCTGGGG CGCATGTGCC AGATGCACCT CAAGTCGGAC<br>1741 AAAGCGCAGA CCAAGCTGCT CATCCTGCAG CAGGCCGTGC AGGTCATCCT GGGGCTGGAG<br>1801 CAGCAGGTGC GAGAGCGGAA CCTGAATCCC AAAGCAGCCT GTTTGAAACG GCGAGAAGAG<br>1861 GAAAAGGTGT CAGGTGTGGT TGGAGACCCC CAGATGGTGC TTTCAGCTCC CCACCCAGGC<br>1921 CTGAGCGAAG CCCACAACCC CGCCGGGCAC ATG
**E47:ΔNLS**	1 ATGAACCAGC CGCAGAGGAT GGCGCCTGTG GGCACAGACA AGGAGCTCAG TGACCTCCTG<br>61 GACTTCAGCA TGATGTTCCC GCTGCCTGTC ACCAACGGGA AGGGCCGGCC CGCCTCCCTG<br>121 GCCGGGGCGC AGTTCGGAGG TTCAGGTCTT GAGGACCGGC CCAGCTCAGG CTCCTGGGGC<br>181 AGCGGCGACC AGAGCAGCTC CTCCTTTGAC CCCAGCCGGA CCTTCAGCGA GGGCACCCAC<br>241 TTCACTGAGT CGCACAGCAG CCTCTCTTCA TCCACATTCC TGGGACCGGG ACTCGGAGGC<br>301 AAGAGCGGTG AGCGGGGCGC CTATGCCTCC TTCGGGAGAG ACGCAGGCGT GGGCGGCCTG<br>361 ACTCAGGCTG GCTTCCTGTC AGGCGAGCTG GCCCTCAACA GCCCCGGGCC CCTGTCCCCT<br>421 TCGGGCATGA AGGGGACCTC CCAGTACTAC CCCTCCTACT CCGGCAGCTC CCGGCGGAGA<br>481 GCGGCAGACG GCAGCCTAGA CACGCAGCCG CCGGGTCTTC CATCCTCGGT GTACCCACCC<br>541 AGCTCAGGTG AGGACTACGG CAGGGATGCC ACCGCCTACC CGTCCGCCAA GACCCCCAGC<br>601 AGCACCTATC CCGCCCCCTT CTACGTGGCA GATGGCAGCC TGCACCCCTC AGCCGAGCTC<br>661 TGGAGTCCCC CGGGCCAGGC GGGCTTCGGG CCCATGCTGG GTGGGGGCTC ATCCCCGCTG<br>721 CCCCTCCCGC CCGGTAGCGG CCCGGTGGGC AGCAGTGGAA GCAGCAGCAC GTTTGGTGGC<br>781 CTGCACCAGC ACGAGCGTAT GGGCTACCAG CTGCATGGAG CAGAGGTGAA CGGTGGGCTC

Table S1. *Cont.*

	841 CCATCTGCAT CCTCCTTCTC CTCAGCCCCC GGAGCCACGT ACGGCGGCGT CTCCAGCCAC
	901 ACGCCGCCTG TCAGCGGGGC CGACAGCCTC CTGGGCTCCC GAGGGACCAC AGCTGGCAGC
	961 TCCGGGGATG CCCTCGGCAA AGCACTGGCC TCGATCTACT CCCCGGATCA CTCAAGCAAT
	1021 AACTTCTCGT CCAGCCCTTC TACCCCCGTG GGCTCCCCCC AGGGCCTGGC AGGAACGTCA
	1081 CAGTGGCCTC GAGCAGGAGC CCCCGGTGCC TTATCGCCCA GCTACGACGG GGGTCTCCAC
	1141 GGCCTGCAGA GTAAGATAGA AGACCACCTG GACGAGGCCA TCCACGTGCT CCGCAGCCAC
	1201 GCCGTGGGCA CAGCCGGTGA CATGCACACG CTGCTGCCTG GCCACGGGGC GCTGGCCTCA
	1261 GGTTTCACCG GCCCCATGTC ACTGGGCGGG CGGCACGCAG GCCTGGTTGG AGGCAGCCAC
	1321 CCCGAGGACG GCCTCGCAGG CAGCACCAGC CTCATGCACA ACCACGCGGC CCTCCCCAGC
	1381 CAGCCAGGCA CCCTCCCTGA CCTGTCTCGG CCTCCCGACT CCTACAGTGG GCTAGGGCGA
	1441 GCAGGTGCCA CGGCGGCCGC CAGCGAGATC AAGCGGGAGG AGAAGGAGGA CGAGGAGAAC
	1501 ACGTCAGCGG CTGACCACTC GGAGGAGGAG AAGAAGGAGC TGAAGGCCCC CCGGGCCCGG
	1561 ACCAGCAGTA CGGACGAGGT GCTGTCCCTG GAGGAGAAAG ACCTGAGGGA CCGGGAGAGG
	1621 CGCATGGCCA ATAACGCGCG GGAGCGGGTG CGCGTGCGGG ATATTAACGA GGCCTTCCGG
	1681 GAGCTGGGGC GCATGTGCCA GATGCACCTC AAGTCGGACA AAGCGCAGAC CAAGCTGCTC
	1741 ATCCTGCAGC AGGCCGTGCA GGTCATCCTG GGGCTGGAGC AGCAGGTGCG AGAGCGGAAC
	1801 CTGAATCCCA AAGCAGCCTG TTTGAAACGG CGAGAAGAGG AAAAGGTGTC AGGTGTGGTT
	1861 GGAGACCCCC AGATGGTGCT TTCAGCTCCC CACCCAGGCC TGAGCGAAGC CCACAACCCC
	1921 GCCGGGCACA TG

Table S2. Primers used for PCR mutagenesis.

Primer ID	Sequence
ATOH7_CACC_for	5′-CAC CAT GAA GTC CTG CAA GC-3′
ATOH7_STOP_rev	5′-TCA TTA CTA GGT GGC CAT CTG G-3′
NLS_ATOH7_for	5′-CAC CAT GCC CAA GAA GAA GCG AAA GGT GAA GTC CTG CAA GCC CAG C-3′
TCF3E47:ΔNLS_for	5'-TAC TCC GGC AGC TCC AAG GTC CCG CCG GGT-3'
TCF3E47:ΔNLS_rev	5'-ACC CGG CGG GAC CTT GGA GCT GCC GGA GTA-3'
